# NLRC5 Exclusively Transactivates MHC Class I and Related Genes through a Distinctive SXY Module

**DOI:** 10.1371/journal.pgen.1005088

**Published:** 2015-03-26

**Authors:** Kristina Ludigs, Queralt Seguín-Estévez, Sylvain Lemeille, Isabel Ferrero, Giorgia Rota, Sonia Chelbi, Chantal Mattmann, H. Robson MacDonald, Walter Reith, Greta Guarda

**Affiliations:** 1 Department of Biochemistry, University of Lausanne, Epalinges, Switzerland; 2 Department of Pathology and Immunology, University of Geneva Medical School, Geneva, Switzerland; 3 Ludwig Center for Cancer Research of the University of Lausanne, Epalinges, Switzerland; The University of Queensland, AUSTRALIA

## Abstract

MHC class II (MHCII) genes are transactivated by the NOD-like receptor (NLR) family member CIITA, which is recruited to SXY enhancers of MHCII promoters via a DNA-binding “enhanceosome” complex. NLRC5, another NLR protein, was recently found to control transcription of MHC class I (MHCI) genes. However, detailed understanding of NLRC5’s target gene specificity and mechanism of action remained lacking. We performed ChIP-sequencing experiments to gain comprehensive information on NLRC5-regulated genes. In addition to classical MHCI genes, we exclusively identified novel targets encoding non-classical MHCI molecules having important functions in immunity and tolerance. ChIP-sequencing performed with *Rfx5^−/−^* cells, which lack the pivotal enhanceosome factor RFX5, demonstrated its strict requirement for NLRC5 recruitment. Accordingly, *Rfx5*-knockout mice phenocopy *Nlrc5* deficiency with respect to defective MHCI expression. Analysis of B cell lines lacking RFX5, RFXAP, or RFXANK further corroborated the importance of the enhanceosome for MHCI expression. Although recruited by common DNA-binding factors, CIITA and NLRC5 exhibit non-redundant functions, shown here using double-deficient *Nlrc5^−/−^CIIta^−/−^* mice. These paradoxical findings were resolved by using a “de novo” motif-discovery approach showing that the SXY consensus sequence occupied by NLRC5 *in vivo* diverges significantly from that occupied by CIITA. These sequence differences were sufficient to determine preferential occupation and transactivation by NLRC5 or CIITA, respectively, and the S box was found to be the essential feature conferring NLRC5 specificity. These results broaden our knowledge on the transcriptional activities of NLRC5 and CIITA, revealing their dependence on shared enhanceosome factors but their recruitment to distinct enhancer motifs *in vivo*. Furthermore, we demonstrated selectivity of NLRC5 for genes encoding MHCI or related proteins, rendering it an attractive target for therapeutic intervention. NLRC5 and CIITA thus emerge as paradigms for a novel class of transcriptional regulators dedicated for transactivating extremely few, phylogenetically related genes.

## Author Summary

Major histocompatibility complex class I (MHCI) molecules are central to immunity and immunological disorders, and constitute a major obstacle in organ transplantation. It is therefore vital to gain insight into the regulation of their expression. NLRC5 was recently found to regulate MHCI gene transcription. However, we lack a thorough understanding of its target gene specificity and mechanism of action. Our work addresses these questions, delineating the unique consensus sequence required for NLRC5 recruitment and pinpointing conserved features conferring its specificity. Furthermore, through genome-wide analyses, we confirm that NLRC5 regulates classical MHCI genes and identify novel target genes, all encoding non-classical MHCI molecules exerting an array of functions in immunity and tolerance. We thereby demonstrate that NLRC5 exclusively transactivates genes of the MHCI pathway, rendering it an attractive target for future therapeutic intervention. The most striking feature of NLRC5 is its restricted and highly focused transcriptional activity, which has been described so far only for one related factor, CIITA. NLRC5 and CIITA therefore emerge as prototypes for a novel kind of extremely specific transcriptional regulator.

## Introduction

Nucleotide-binding oligomerization domain (NOD)-like receptors (NLRs) constitute a family of innate immune receptors involved mainly in inflammatory responses and cell death. The NLR family member CIITA instead functions as the master transcriptional regulator of major histocompatibility complex (MHC) class II (MHCII) genes, and mutations in the *CIITA* gene lead to severe immunodeficiency [1]. Recently, NLR caspase recruitment domain containing protein 5 (NLRC5) was shown to regulate transcription of MHC class I (MHCI) genes, primarily in lymphocytes, where it is highly expressed [2,3,4,5,6]. Overexpression of NLRC5 was initially found to increase mRNA levels for genes encoding human MHCI molecules and proteins functioning in the MHCI-mediated antigen presentation pathway, including beta-2-microglobulin (B2M), transporter associated with antigen processing 1 (TAP1) and the proteasome subunit beta type-9 (PSMB9) [3]. Four independently generated *Nlrc5*-knockout mice subsequently established that NLRC5 regulates the expression of *B2m*, *Tap1*, *Psmb9*, classical MHCI genes (*H2-K*, *H2-D*), and the non-classical MHCI gene *H2-M3* [2,4,5,7]. Finally, *in vivo* promoter occupancy by NLRC5 was demonstrated only for human *HLA-A* and *HLA-B*, and mouse *H2-K*, *H2-D*, and *B2m* [2,3].

CIITA-dependent transactivation of MHCII genes requires the SXY motif, a conserved enhancer found in all MHCII promoters. DNA-binding factors recognizing this element form an “enhanceosome” complex that serves as a platform for the recruitment of CIITA [1]. The X-binding regulatory factor X (RFX) complex is essential for enhanceosome assembly and CIITA recruitment. A similar SXY motif is found in MHCI gene promoters, together with more distal regulatory elements, and has been implicated in NLRC5-mediated transactivation [8,9]. Enforced expression of the RFX5, RFXAP, and RFXANK subunits of RFX potentiated NLRC5-driven MHCI transcription, and interaction between NLRC5 and overexpressed RFXANK was observed [8].

The shared use of enhanceosome factors by CIITA and NLRC5 suggests that these NLRs might fulfill partially redundant functions, a hypothesis that has not been tested *in vivo*. The relevance of endogenous enhanceosome factors for NLRC5-mediated MHCI-transactivation has also not been assessed. Furthermore, a comprehensive set of genes regulated directly by NLRC5 has not been defined. Finally, most NLRC5 target genes are encoded within the MHCI locus, raising the question of whether NLRC5 specifically regulates each one individually or if it instead establishes an open chromatin conformation at the entire locus. To address these questions we compared CIITA and NLRC5-regulated gene expression in various cell types from *Rfx5* ^*−/−*^, *Nlrc5* ^*−/−*^, *CIIta* ^*−/−*^ and *CIIta* ^*−/−*^ *Nlrc5* ^*−/−*^ mice, as well as in CIITA and RFX-deficient B cell lines, and screened for NLRC5 target genes by means of chromatin immunoprecipitation sequencing (ChIP-seq) experiments performed with T cells from control, *Nlrc5* ^−/−^, and *Rfx5* ^−/−^ mice.

We found that NLRC5 is remarkably dedicated for a small set of related genes: it selectively occupies the promoters of genes coding for MHCI or related proteins, and identified the non-classical MHCI genes *H2-Q4*, *H2-Q6/7*, and *H2-T10/22* as novel NLRC5-regulated genes. Analysis of NLRC5-binding in *Rfx5*-deficient cells demonstrated that Rfx5 is essential for promoter occupancy by NLRC5. Data generated in B cell lines carrying mutations in RFX5, RFXAP, and RFXANK also indicated a key requirement for the enhanceosome in MHCI transactivation. However, despite their recruitment by common factors, analysis of single (*CIIta* ^*−/−*^, *Nlrc5* ^*−/−*^) and double deficient (*CIIta* ^*−/−*^ *Nlrc5* ^*−/−*^) mice revealed that CIITA and NLRC5 are highly specific for distinct sets of genes. Identification of the consensus sequence occupied *in vivo* by NLRC5 highlighted unique features that were shown to be responsible for NLRC5 specificity.

## Results

### *Nlrc5* ^−/−^ and *Rfx5* ^−/−^ mice exhibit similar defects in MHCI expression

**Fig 1:**
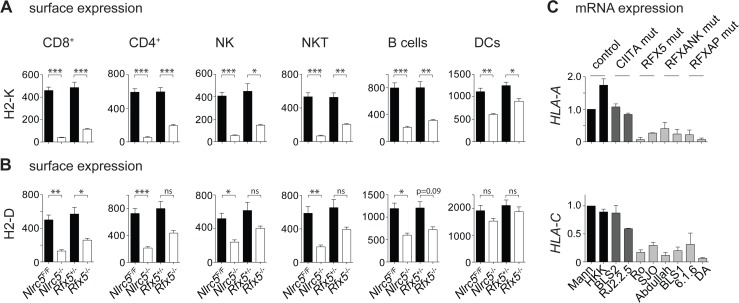
MHCI expression is strongly reduced in *Nlrc5* ^−/−^ and *Rfx5* ^−/−^ lymphocytes. (A and B) MHCI expression on the indicated splenic cells was analyzed in *Nlrc5* ^*F/F*^ (n = 9), *Nlrc5* ^*−/−*^ (n = 9), *Rfx5* ^*+/-*^ (n = 10), and *Rfx5* ^*−/−*^ (n = 8) mice. Graphs illustrate geometric mean fluorescence intensities (MFIs) of H2-K (A) and H2-D (B) for CD8^+^ T cells (gated as CD3^+^CD8^+^), CD4^+^ T cells (gated as CD3^+^CD4^+^), NK cells (gated as NK1.1^+^CD3^-^), NKT cells (gated as NK1.1^+^CD3^+^), B cells (gated as CD19^+^), and DCs (gated as CD11c^hi^CD11b^int-hi^). Results represent the mean ± SEM from two pooled experiments. Statistical significance was calculated using an unpaired Student’s *t*-test, two-tailed; adjustment was made using a Bonferroni correction over 12 samples (A and B). (C) *HLA-A* and *HLA-C* mRNAs were quantified by qRT-PCR in WT (Mann, HHK), CIITA-deficient (BLS2, RJ2.2.5), RFX5-deficient (Ro, SJO), RFXANK-deficient (BLS1, Abdulla), and RFXAP-deficient (6.1.6, DA) B cell lines. Results were normalized using *ACTB*, expressed relative to Mann and represent the mean ± SD derived from three independent experiments.

*Rfx5*-deficient mice were exploited to assess the role of the enhanceosome factor Rfx5 in MHCI expression. Analysis of H2-K cell-surface expression by flow cytometry in various immune cell subsets derived from *Rfx5* ^*+/-*^ and *Rfx5* ^*−/−*^ littermates demonstrated that *Rfx5*-deficiency led to a strong decrease in MHCI expression on T cells, NK cells, and NKT cells, a marked reduction on B cells, and a more modest decrease on dendritic cells (DCs) (Fig. 1A). A similar trend, albeit less strong, was observed for *H2-D* (Fig. 1B). This phenotype was strikingly similar to that of *Nlrc5*-deficient cells (Fig. 1A and B). However, the defect in MHCI expression observed in the absence of *Rfx5* was always slightly less profound as compared to that in *Nlrc5*-deficient cells, suggesting the existence of mechanisms capable of compensating partially for the deficiency in Rfx5.

We also measured MHCI mRNA expression by quantitative real-time RT-PCR (qRT-PCR) in *in vitro*-generated B cell mutants and B cell lines derived from bare lymphocyte syndrome (BLS) patients carrying inactivating mutations in CIITA, RFX5, RFXAP, and RFXANK. These experiments underlined the importance of RFX factors for MHCI expression (Fig. 1C) [10,11]. Collectively, these results support a role for the enhanceosome in the recruitment and transcriptional activity of NLRC5, in both human and mouse cells.

### NLRC5 and CIITA have non-redundant functions

**Fig 2:**
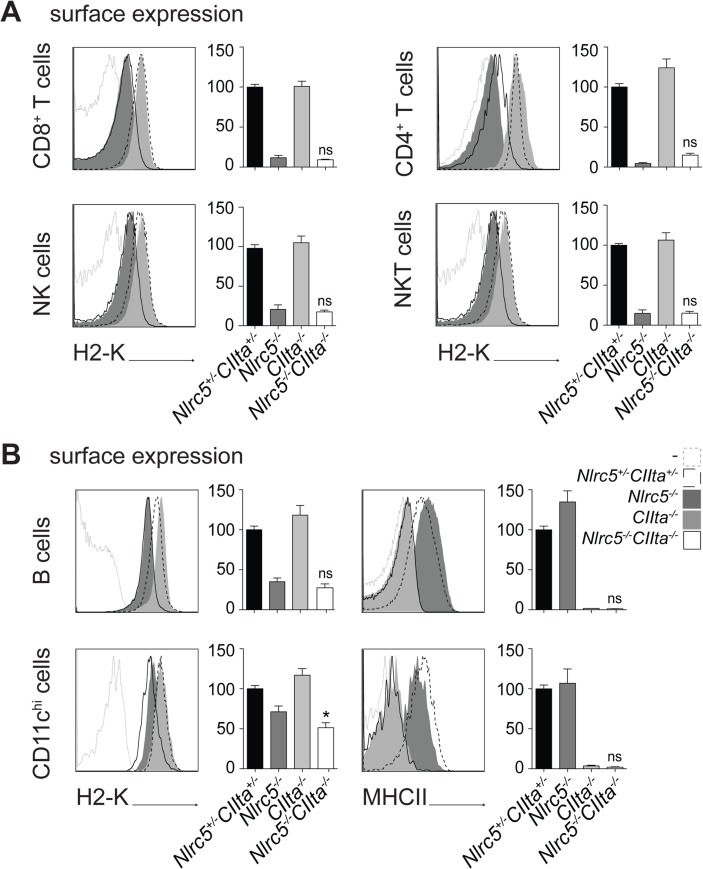
NLRC5 and CIITA exhibit non-redundant functions. (A and B) MHCI and MHCII expression were assessed in the indicated splenic cells from *Nlrc5* ^*+/−*^ *CIIta* ^*+/−*^, *Nlrc5* ^*−/−*^, *CIIta* ^*−/−*^, and *Nlrc5* ^*−/−*^ *CIIta* ^*−/−*^ mice. Histogram overlays show H2-K or MHCII expression for control (dashed line), *Nlrc5* ^−/−^ (filled dark grey), *CIIta* ^−/−^ (filled light grey), and double-deficient (solid line) mice. Graphs depict the MFIs of H2-K for CD8^+^ T cells (gated as CD3^+^CD8^+^), CD4^+^ T cells (gated as CD3^+^CD4^+^), NK cells (gated as NK1.1^+^CD3^-^), and NKT cells (gated as NK1.1^+^CD3^+^) (A), or B cells (gated as CD19^+^) and DCs (gated as CD11c^hi^CD11b^int-hi^) (B) in control (n = 9), *Nlrc5* ^−/−^ (n = 10), *CIIta* ^−/−^ (n = 9), and double-deficient (n = 8) mice (A and B). For B cells and DCs, graphs also show the MFI of MHCII. MFIs of control mice were set at 100%. (A and B) Results depict the mean ± SEM from three pooled experiments. Statistical significance of the differences between multiple groups was analyzed by 2-way ANOVA adjusted by Bonferroni correction over 6 (H2-K) or 2 (MHCII) samples, and is indicated only for differences between single and double-deficient groups (i.e., the interaction between the two groups).

The fact that both NLRC5 and CIITA dock to similar SXY modules via shared enhanceosome factors raised the question of whether or not these two NLRs are overlapping in their transactivation role. That the two factors might exhibit partial redundancy in MHCI-transactivation was suggested by the findings that decreased MHCI expression caused by NLRC5-deficiency is more pronounced in T, NK, and NKT lymphocytes, which do not express CIITA, than in antigen-presenting cells (APCs) and thymic epithelial cells (TECs) [2], which express high levels of CIITA. Previous studies had also suggested that CIITA can stimulate MHCI transcription [10,11] and that MHCI promoters are occupied by CIITA in APCs [12,13]. We therefore generated double-deficient *Nlrc5* ^*−/−*^ *CIIta* ^*−/−*^ mice, and studied MHC expression in different immune cell subsets by flow cytometry. Concomitant ablation of *Nlrc5* and *CIIta* did not substantially reduce H2-K and H2-D levels compared to single *Nlrc5*-deficiency, neither in any hematopoietic cell type analyzed nor in medullary TECs (Figs. 2A and B, S1A and S1B), although a minor but significant decrease was observed for H2-K in DCs. Accordingly, frequencies of peripheral CD8^+^ T cells, which require MHCI for their development and maintenance, were not decreased more strongly in *Nlrc5* ^*−/−*^ *CIIta* ^*−/−*^ mice than in *Nlrc5* ^*−/−*^ mice (S1C Fig.). MHCII expression was not reduced further in APCs from double-knockout animals, being already at negligible levels in single *CIIta*-deficient cells (Fig. 2B). These data indicate that NLRC5 and CIITA are highly specific for transactivating different sets of genes, even though they rely on common DNA binding factors for their recruitment.

### NLRC5 binds to promoters of known and newly identified MHCI genes in an Rfx5-dependent manner

To gain a comprehensive view of genes regulated transcriptionally by NLRC5, we performed ChIP-seq experiments in T cells, which express NLRC5 abundantly and exhibit a dramatic defect in MHCI levels upon its ablation. Chromatin was extracted from T cells derived from control (WT and *Nlrc5* ^*F/F*^), *Nlrc5* ^*−/−*^, and *Rfx5* ^−/−^ mice. NLRC5-bound chromatin was enriched by ChIP and submitted to deep sequencing.

**Fig 3:**
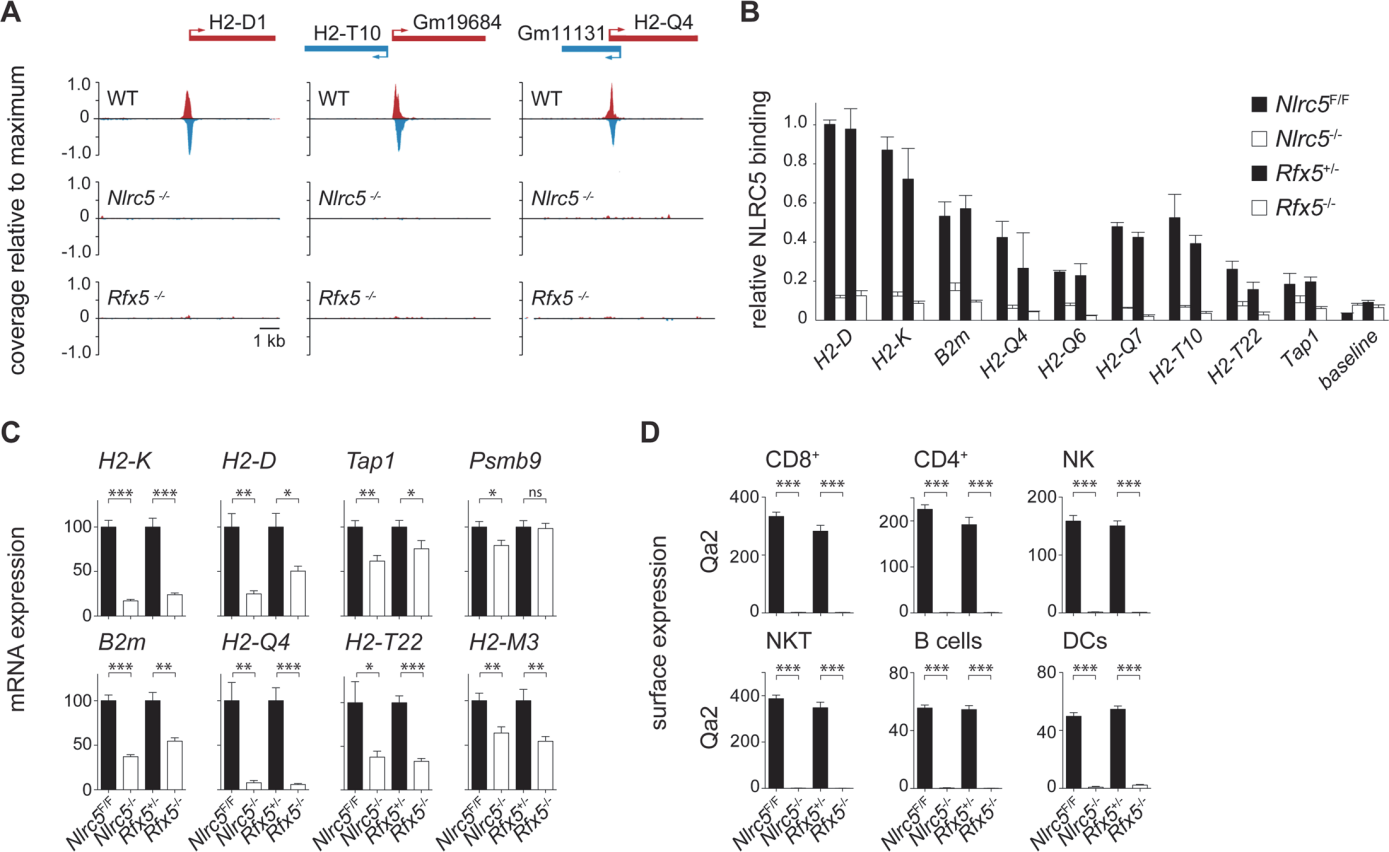
Identification of NLRC5 targets. (A) NLRC5-ChIPseq tracks are shown for *H2-D1*, *H2-T10/Gm19684* and *Gm11131/H2-Q4*. The tracks depict reads mapping to regions spanning between 5kb upstream and 5kb downstream of the peaks. After normalization as rpm (reads per millions), read coverage was expressed relative to the maximal value observed in the region. TSSs are positioned as annotated in ENSEMBL (*H2-T10*, *H2-Q4*) or Refseq (*H2-D1*). (B) Binding of NLRC5 was assessed by quantitative ChIP experiments performed with CD8^+^ T cells from *Nlrc5* ^*F/F*^, *Nlrc5* ^*−/−*^, *Rfx5* ^*+/-*^, *and Rfx5* ^*−/−*^ mice. Results are expressed relative to binding of NLRC5 to the *H2-D* promoter in *Nlrc5* ^*F/F*^ cells, and depict the mean ± SD derived from three technical replicates and are representative of two independent experiments. (C) mRNAs for the indicated genes were quantified relative to *Hprt* mRNA in CD8^+^ T cells purified from a pool of 4–5 mice of each indicated genotype. Values for *Nlrc5* ^−/−^ and *Rfx5* ^−/−^ cells are expressed as percentage of *Nlrc5* ^*F/F*^ and *Rfx5* ^+/-^, respectively. Data represent mean ± SD of technical triplicates, and are representative of at least two independent experiments. Statistical significance was calculated with an unpaired Student’s *t*-test, two-tailed; adjustment was made using a Bonferroni correction over 2 samples. (D) MFI for Qa2 was analyzed in the indicated cell subsets from *Nlrc5* ^*F/F*^ (n = 9), *Nlrc5* ^−/−^ (n = 9), *Rfx5* ^+/-^ (n = 10), and *Rfx5* ^−/−^ (n = 8) mice. Specific cell populations were gated on CD3^+^CD8^+^ for CD8^+^ T cells, CD3^+^CD4^+^ for CD4^+^ T cells, NK1.1^+^CD3^-^ for NK cells, NK1.1^+^CD3^+^ for NKT cells, CD19^+^ for B cells, and CD11c^hi^CD11b^int-hi^ for DCs. Results represent the mean ± SEM derived from two pooled experiments. Statistical significance was calculated with an unpaired Student’s *t*-test, two-tailed; adjustment is made using a Bonferroni correction over 12 samples.

**Fig 4:**
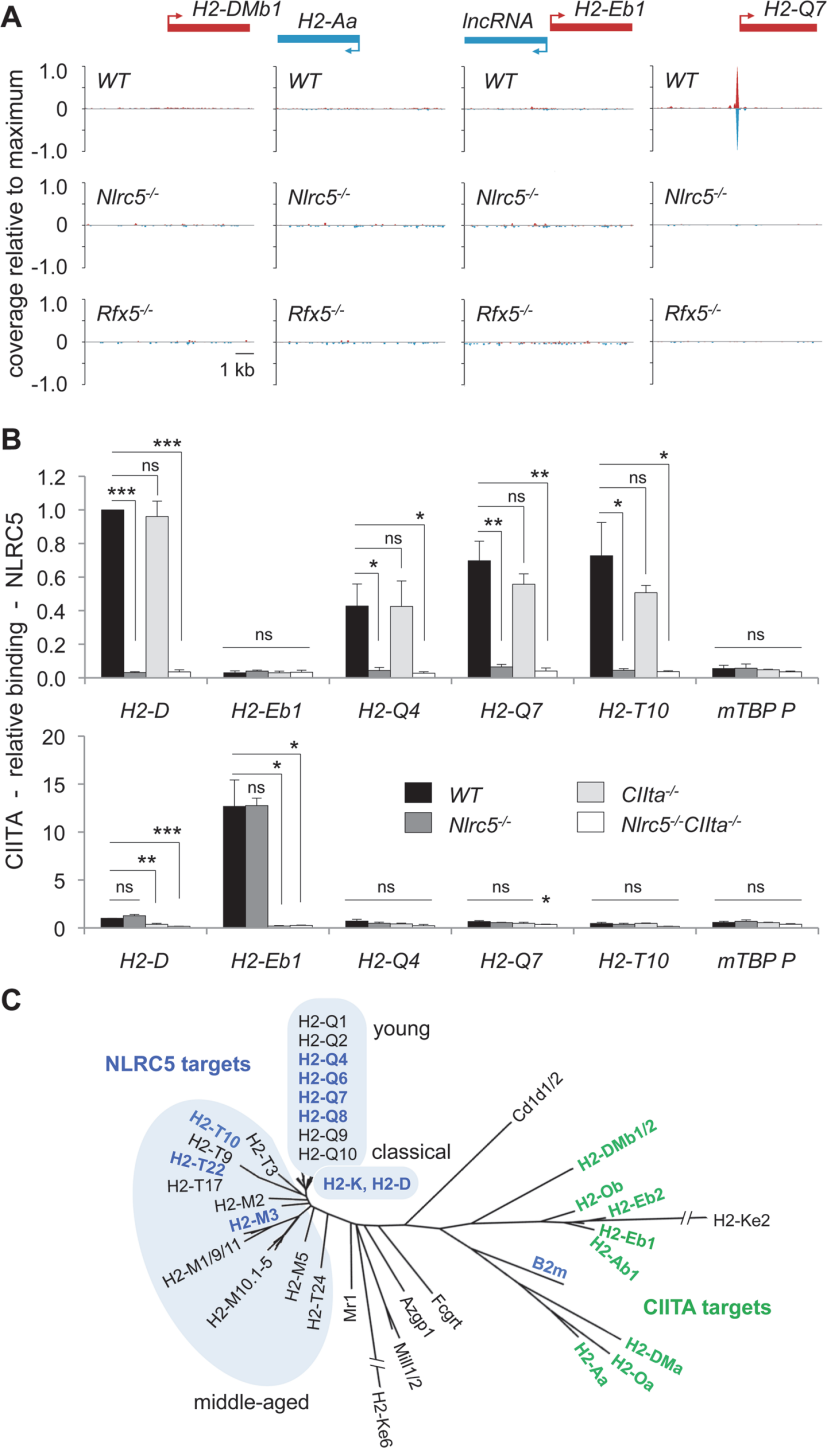
NLRC5 selectively occupies MHCI gene promoters. (A) NLRC5-ChIPseq tracks are shown for the CIITA targets *H2-DMb1*, *H2-Aa* and *IncRNA/H2-Eb1*. The tracks depict reads mapping to regions spanning between 5kb upstream and 5kb downstream of the TSS. After normalization as rpm (reads per million), read coverage was expressed relative to the average value observed for all NLRC5-binding peaks. TSSs are positioned as annotated in ENSEMBL. (B) Antibodies specific for NLRC5 and CIITA were used to immunoprecipitate cross-linked chromatin fragments derived from *Nlrc5* ^*+/-*^ *CIIta* ^*+/-*^, *Nlrc5* ^*−/−*^, *CIIta* ^*−/−*^, and *Nlrc5* ^*−/−*^ *CIIta* ^*−/−*^ B cells. Immunoprecipitates were analyzed by quantitative PCR for the abundance of promoter sequences from the indicated genes. Relative promoter binding is shown. The average ± SEM of three experiments are depicted. Statistical significance was calculated using an unpaired Student’s *t*-test. (C) A phylogenetic tree is shown for classical and non-classical MHCI and MHCII genes. Genes regulated by NLRC5 and those regulated by CIITA are indicated in blue and green font, respectively. Clusters of classical, young and middle-aged MHCI genes are highlighted.

As previously observed for CIITA [12], and in sharp contrast to most other transcription factors, which typically occupy large numbers of sites in the genome [14], only a restricted number of NLRC5-occupied sites were detected. A total of only 11 NLRC5-binding sites were present in control (WT and/or *Nlrc5* ^*F/F*^) cells but absent in *Nlrc5* ^*−/−*^ cells (Table 1, Figs. 3A, 4A, S2A). Of the NLRC5-occupied sites, 9 resided in the vicinity (-500 to +50) of the transcription start sites (TSSs) of 12 genes. The number of genes exceeds the number of peaks because 3 peaks lie between two closely spaced genes present in divergent orientations. Peaks at these promoter sites were all absent in *Rfx5* ^*−/−*^ cells (Table 1, Figs. 3A, 4A, S2A). The remaining two NLRC5-occupied sites were situated far from known promoters on chromosomes 1 and 15 and were not *Rfx5*-dependent (Table 1).

**Table 1:** NLRC5-occupied sites and target genes.

Gene	Gene ID	Peak identification	Peak coordinates ^1^	TSS position (orientation)	RFX5 dependent	ChIP validation	Expression validation	NLRC5 module
***B2m***	ENSMUSG00000060802	peak calling	chr2:122147175-122148130	chr2:122147686 (+)	+	+	+ ^3^	+
***H2-D1***	ENSMUSG00000073411	peak calling	chr17:35262463-35263394	chr17:35262730 (+)	+	+	+ ^3^ ^,^ ^4^	+
***H2-K1***	ENSMUSG00000061232	peak calling	chr17:34000049-34000768	chr17:34000333 (-)	+	+	+ ^3^ ^,^ ^4^	+
***H2-Q4***	ENSMUSG00000035929	peak calling	chr17:35379037-35380004	chr17:35379617 (+)	+	+	+ ^3^	+
***H2-Q6***	ENSMUSG00000073409	peak calling	chr17:35424724-35424884	chr17:35424850 (+)	+	+	+ ^4^	+
***H2-Q7***	ENSMUSG00000060550	peak calling	chr17:35438815-35439225	chr17:35439155 (+)	+	+	+ ^4^	+
***H2-T10*** ^5^	ENSMUSG00000079491	peak calling	chr17:36121380-36122293	chr17:36121465 (-)	+	+	nd	+
***Gm19684*** ^5^	ENSMUSG00000092277	peak calling	chr17:36121380-36122293	chr17:36121678 (+)	+	+	nd	+
***H2-T22*** ^6^	ENSMUSG00000056116	peak calling	chr17:36042691-36043453	chr17:36042747 (-)	+	+	+ ^3^	+
***Gm6034*** ^6^	ENSMUSG00000073407	peak calling	chr17:36042691-36043453	chr17:36042961(+)	+	+	nd	+
***Tap1*** ^7^	ENSMUSG00000037321	peak calling	chr17:34187312-34187863	chr17:34187553 (+)	+	+	+ ^3^	+
***Psmb9*** ^7^	ENSMUSG00000096727	peak calling	chr17:34187312-34187863	chr17:34187764 (-)	+	+	+ ^3^	+
***H2-M3***	ENSMUSG00000016206	visual ^2^	na	chr17:37270220 (+)	+	nd	+ ^3^	-
***Aida*** ^8^	ENSMUSG00000042901	peak calling	chr1:183299156-183299382	chr1:183297060 (+)	-	nd	nd	-
***Ly6c2*** ^8^	ENSMUSG00000022584	peak calling	chr15:75085781-75085890	chr15:75111584 (-)	-	nd	nd	-

^1^ determined by peak calling

^2^ identified by visual inspection of ChIP-seq tracks

^3^ qRT-PCR

^4^ flow cytometry

na not applicable

nd not determined

^5^ *H2-T10* and *Gm6034* share the same peak

^6^ *H2-T22* and *Gm19684* share the same peak

^7^ *Tap1* and *Psmb9* share the same peak

^8^ gene situated closest to peak

Genes containing NLRC5-occupied sites in their promoter regions included genes previously suggested to be regulated by NLRC5 (*H2-K*, *H2-D*, *B2m*, *Psmb9*, and *Tap1*), validating the quality of the ChIP-seq analysis (Table 1, Figs. 3A, S2A). In addition, novel target genes were identified (Table 1, Figs. 3A, 4A). Five of these are non-classical MHCI genes (*H2-Q4*, *H2-Q6*, *H2-Q7*, *H2-T10*, and *H2-T22*). Two are predicted genes of unknown function (*Gm19684*, *Gm6034*) situated in the reverse orientation immediately upstream of H2-T10 and H2-T22 (Fig. 3A and Table 1).

To ensure that the peak calling procedure had not missed binding sites in other MHC genes, the entire MHC locus was scanned visually for potential binding sites. This identified only one additional non-classical MHCI promoter (*H2-M3*) (Table 1). The latter was missed by the peak-calling algorithm because of its low intensity. In contrast to CIITA [15], no NLRC5-occupied intergenic enhancers were identified in the MHC locus.

Most NLRC5 targets were validated by classical quantitative ChIP experiments (Fig. 3B). To investigate the relevance of NLRC5 and Rfx5 for transactivation of the target genes identified by ChIP-seq, mRNA expression was quantified by qRT-PCR in control, *Nlrc5* ^−/−^, and *Rfx5* ^−/−^ CD8^+^ T cells (Fig. 3C). Transcript abundance of tested NLRC5 targets was reduced in the absence of either *Nlrc5* or *Rfx5*, with the exception of *Psmb9*, whose expression was not altered in the absence of *Rfx5*. High homology among non-classical MHCI genes did not allow quantification of *H2-T10*, *H2-Q6*, and *H2-Q7* by qRT-PCR. However, we measured expression of the Qa2 antigen (encompassing H2-Q6/7/8/9) by flow cytometry and observed a virtually complete loss in all tested cell types in the absence of *Nlrc5* or *Rfx5* (Fig. 3D). Collectively, these results provide evidence for the critical importance of Rfx5 in recruiting NLRC5 and for the contribution of the Rfx5-NLRC5 axis in activating most of the identified target genes.

### NLRC5 and CIITA occupy distinct promoters

Although NLRC5 and CIITA are recruited by common enhanceosome factors, NLRC5-binding was not observed at the promoters of any MHCII genes (Fig. 4A). As the ChIP-sequencing was performed in T lymphocytes, which do not express MHCII genes, we reasoned that an inaccessible chromatin conformation might prevent NLRC5-binding to MHCII promoters in these cells. We therefore immunoprecipitated NLRC5 and CIITA bound chromatin from control, *Nlrc5* ^−/−^, *CIIta* ^−/−^, and *Nlrc5* ^−/−^ *CIIta* ^−/−^ B cells, which express high levels of CIITA and MHCII. Quantitative ChIP analysis confirmed that NLRC5 binding was observed at classical and non-classical MHCI promoters but not at the prototypical *H2-E* MHCII promoter (Fig. 4B). As in T cells, NLRC5 recruitment was dependent on Rfx5 in B cells (S2B Fig.). CIITA binding was evident at the *H2-E* promoter but not at any of the NLRC5 targets tested (Fig. 4B). These results are consistent with our MHC expression data showing non-redundant functions of NLRC5 and CIITA (Fig. 2).

These results emphasize the striking specificity of NLRC5 and CIITA for phylogenetically related but distinct sets of genes (Fig. 4C). Interestingly, NLRC5-controlled genes encode classical and evolutionarily “middle-aged” and “young” non-classical MHCI molecules [16], with the exception of *B2m*, which clusters together with MHCII molecules. This suggests that divergent evolution underlies the differentiation of NLRC5 function and specificity.

### Identification of a unique consensus motif for NLRC5 binding

**Fig 5:**
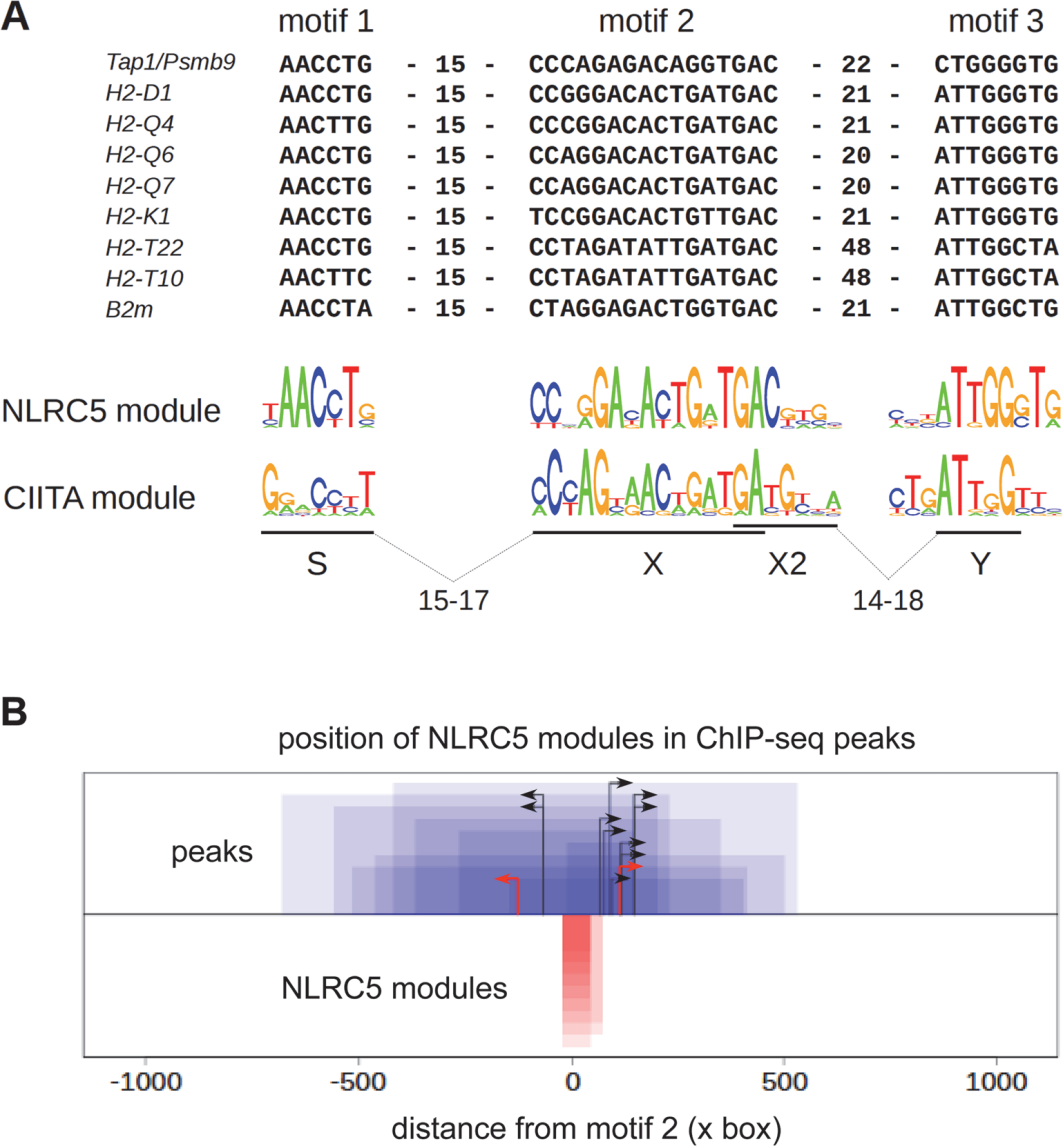
Identification of a sequence module for selective NLRC5 recruitment. (A) Alignment of sequence motifs situated within NLRC5-occupied peaks found in promoter regions of the indicated genes. S-X and X-Y distance constraints used for identifying S and Y boxes were chosen such that these motifs should be situated within 60 bp windows upstream or downstream of the center of the X box. Distances (bp) between motifs are indicated. The sequence logo for the consensus NLRC5-module is shown below the alignment and is compared with that previously defined for human CIITA. (B) NLRC5-binding peaks (blue boxes) were oriented according to the direction of their SXY modules (red boxes). The 0 position on the x-axis corresponds to the start of the X box. Peaks and modules are paired by their heights on the y-axis. Black arrows represent TSS positions annotated in ENSEMBL. For two genes (*H2-D* and *Psmb9*) the predicted TSSs from Refseq (red arrows) are shown because the TSSs annotated in ENSEMBL would be situated upstream of the SXY module (see S2 Table).

A consensus sequence motif with similarity to the X box (Fig. 5A) was derived from promoter-associated NLRC5-occupied sequences using an unbiased motif discovery approach. As organization of the S, X, and Y elements in human MHCII promoters is tightly constrained with respect to their spacing [15,17], we searched for S and Y motifs located at the expected distance ranges from the X box (Fig. 5A). For most NLRC5 targets, we identified S and Y elements situated at distances within 16 and 20–22 base pairs, respectively, from the X box (Figs. 5A, S3, and S4A). Y motifs were not found at 20–22 base-pair distances from the X box in the *H2-T10* and *H2-T22* promoters. We therefore performed a less stringent search for S and Y motifs situated at more variable distances upstream and downstream of the X box (S5 Fig.). This search revealed the presence of Y motifs situated 48 base pairs downstream of the X box in the H2-T promoters (Figs. 5A, S3, S4B, and S5). It also identified sequences exhibiting similarity to the S box situated 45 base pairs upstream of the X box in these genes (S5 Fig.). Intriguingly, this motif contains a Y sequence, which might influence expression of *H2-T10* and *H2-T22* genes. At all NLRC5 targets, the identified SXY modules were situated upstream of the TSS, near the center of the NLRC5-binding peak (Fig. 5B).

Irrespectively of the two approaches used for their identification, the SXY module defined for NLRC5-binding diverges substantially from that observed for CIITA, particularly at the level of the S box and at selected positions within the X box (Figs. 5A and S5).

A scan of the entire genome with the consensus motifs defined in Figs. 5A and S5 identified 15 and 173 putative matches, respectively. This indicates that spacing is a critical determinant for NLRC5 binding (S4 Fig.), since relaxing the spacing constraint leads to a larger number of predicted consensus sequences that are not actually occupied by NLRC5 (S4B Fig.). Among the hits obtained with the more stringent screen, 11 were in found in the vicinity of TSSs (S1 Table). These matches corresponded to the promoter-associated NLRC5-occupied sites and no MHCII genes or other CIITA-regulated genes were identified, underscoring the specificity of the consensus motif for NLRC5-recruitment.

### The S box sequence determines NLRC5-specific transactivation

**Fig 6:**
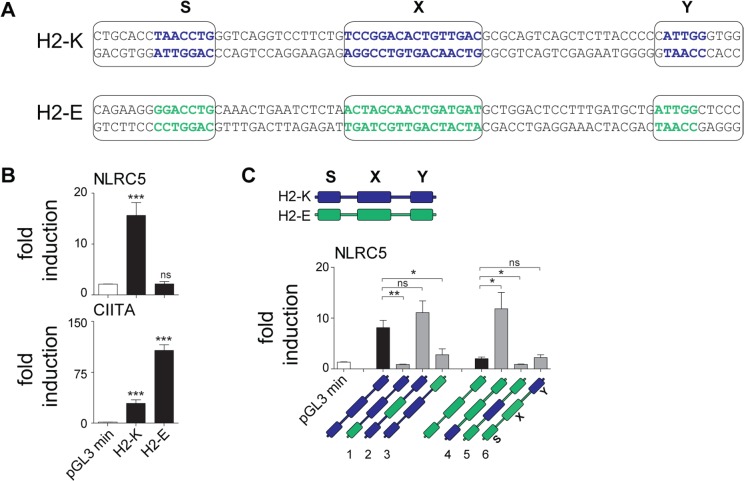
The S box sequence is required for NLRC5-mediated transactivation. (A) Alignment of SXY regions of *H2-K* and *H2-Eb (H2-E)*. Bold letters indicate the most conserved sequences in the S, X, and Y motifs; boxes define the regions that were used to generate hybrid promoters 1–6 (see Material and Methods) shown in C. (B) Luciferase reporter gene analyses were performed in HEK293T cells co-transfected with the *H2-K* or *H2-Eb* reporter constructs and either empty vector (e.v.) or expression vectors encoding NLRC5 or CIITA. Data represent mean ± SD of technical triplicates expressed as fold induction over e.v. and are representative of at least three experiments. Statistical significance was calculated using an unpaired Student’s *t*-test, two-tailed. (C) Luciferase reporter gene analyses were performed in HEK293T cells co-transfected with empty or NLRC5-encoding expression vectors and the WT (*H2-K*, *H2-E*) or hybrid (H2-K/E 1–6) promoter constructs depicted schematically below: *H2-K* and *H2-Eb* derived sequences are represented by blue and green, respectively. Data are expressed as fold induction over e.v. and represent mean ± SEM of four independent experiments. Statistical significance was calculated using an unpaired Student’s *t*-test, two-tailed.

To investigate whether differences between the SXY modules bound by NLRC5 and CIITA were sufficient to confer transactivating specificity, we cloned the SXY regions of *H2-K* and *H2-Eb* into reporter plasmids. These two SXY modules were chosen based on their high similarity to the consensus motifs defined for NLRC5 and CIITA, respectively (Fig. 6A). NLRC5 exclusively transactivated the *H2-K* construct, whereas CIITA preferentially activated the *H2-Eb* construct (Fig. 6B). These results provided direct evidence that the SXY region dictates the differential promoter specificities of NLRC5 and CIITA.

To pinpoint the elements conferring NLRC5 specificity, we generated a series of hybrid promoters in which individual S, X, and Y boxes of *H2-K* were replaced with the corresponding ones from *H2-Eb* and *vice versa (* Fig. 6C). Despite differences in the X box consensus sequences defined for NLRC5 and CIITA (Fig. 5A), reporter assays performed with the hybrid promoters indicated that the X boxes from *H2-K* and *H2-Eb* were equally efficient at supporting NLRC5-mediated transactivation (Fig. 6C). The Y box of *H2-K* partially contributed to NLRC5 activity but was not sufficient *per se* (Fig. 6C). In contrast, the *H2-K* S motif proved to be critical for driving NLRC5-mediated activity, as its replacement with the S box of *H2-Eb* was sufficient to abolish transactivation (Fig. 6C). Furthermore, this element was sufficient for promoting NLRC5-induced transcription when placed into the *H2-Eb* reporter backbone (Fig. 6C). These results show that the unique S box motif found in the promoters of NLRC5-regulated genes is the major determinant for guiding selective gene activation by NLRC5.

## Discussion

Our understanding of NLRC5’s function as a transcriptional regulator of MHCI genes has progressed rapidly during recent years; yet several fundamental aspects remained unexplored. Here, we provide a comprehensive analysis of NLRC5-regulated genes in T cells, leading to the identification of novel target genes and gaining new insights into the molecular mechanisms of NLRC5 recruitment to specific promoters.

Interestingly, NLRC5-transactivated MHCI genes encode classical and evolutionarily “middle-aged” and “young” non-classical MHCI molecules, which generally support T cell receptor engagement and NK cell inhibition [16]. The expression of non-classical MHCI molecules, such as the novel target Qa2, have been shown to be important for the selection of non-conventional T cell subsets and in the development of the preimplantation embryo [18,19,20]. H2-T10 and H2-T22 have been implicated in the selection of gamma-delta T cells with immunoregulatory functions [21,22]. Since the selection of unconventional T cell subsets is mainly driven by hematopoietic cells, and could occur through T cell-T cell interactions, our data generated in T lymphocytes might be particularly relevant for this process [16,23,24]. Taken together, it appears that NLRC5 function has specifically co-evolved with the needs for MHCI-restricted antigen-presentation to conventional or non-conventional T cell subsets, and with NK cell education, suggesting the need to take a closer look at the role of NLRC5 in the development of these subsets.

We provide evidence that Rfx5 serves as a key mediator of NLRC5 binding to the promoter of its target genes, as its absence abolished NLRC5 recruitment to all target genes. Together with evidence that RFX5, RFXAP and RFXANK contribute to HLA class I transcription in human B cells, our findings unambiguously clarify the molecular nature of BLS type III disorders, which are characterized by defects in both MHCI and MHCII expression [8,9,10,11].

Analysis of double-deficient mice demonstrated that CIITA and NLRC5 regulate distinct sets of genes despite the fact that they use common enhanceosome factors and similar promoter sequences. This surprising situation raises the question as to how specificity is achieved. ChIP-seq analysis allowed us to detail the preferential promoter module occupied by NLRC5. Most prominently, selected positions within the X box and the remarkably conserved S box emerged as key features associated with NLRC5 recruitment, thereby distinguishing the SXY region recognized by NLRC5 from that occupied by CIITA. This is consistent with the results of reporter gene assays suggesting that the S box is required for NLRC5-mediated transactivation [8]. We demonstrate here that the distinctive S motif found in the promoters of NLRC5-occupied genes is essential for conferring the transactivation specificity of NLRC5, and that its replacement by the analogous S motif of CIITA-occupied promoters abrogates NLRC5 transactivation. This critical role of the S box suggests that the SXY module occupied by NLRC5 promotes the assembly of an enhanceosome complex differing from that required for the recruitment of CIITA, although the two complexes do share certain DNA-binding proteins. In this respect it should be mentioned that the S box-binding factors remain to be identified and could differ between NLRC5 and CIITA regulated genes.

Polymorphisms within the MHCI locus have been associated with infectious and autoimmune diseases. In many cases, the determining parameter is the MHCI haplotype, as different alleles can present different peptide repertoires. However, it has recently been suggested that various alleles can also be expressed at dissimilar levels, and that their abundance shows significant associations with disease outcomes, as in the case of human immunodeficiency virus infection and Crohn’s disease [25]. Given the fact that the SXY module is conserved between mouse and humans [26], it will be important to establish whether promoter variants of alleles associated with immunological disorders are differentially transactivated by NLRC5. Such correlations could be of high medical relevance as predictive or prognostic markers in selected immunological diseases.

The newly identified NLRC5 target genes encode non-classical MHCI molecules, emphasizing the remarkable selectivity of this NLR for regulating the MHCI system. This renders NLRC5 an attractive candidate for therapeutic intervention aimed at modulating MHCI expression. The high specificity of NLRC5 for a small number of phylogenetically related MHCI genes is strikingly similar to that of CIITA, for which ChIP-on-microarray experiments have revealed high selectivity for genes involved in MHCII-mediated antigen presentation [12]. Although recent ChIP-seq experiments have suggested that there are other CIITA-occupied sites in the genome [27], their functional relevance remains to be demonstrated. The extremely focused activity of NLRC5 and CIITA sets them apart from other transcription factors and transcriptional coactivators, which typically regulate hundreds or thousands of genes and exhibit much more diverse and pleiotropic functions; these two NLRs are instead specialized for the expression of only few phylogenetically and/or functionally related genes, representing a novel type of highly dedicated transcriptional regulator.

## Materials and Methods

### Mice

Mice were treated in accordance with the Swiss Federal Veterinary Office guidelines. *Nlrc5* ^*F/F*^, *Nlrc5* ^*−/−*^ [2], *CIIta* ^*−/−*^ [28], and C57BL/6 control mice, all on a C57BL/6 (H2^b^) background, were bred at the animal facility of the University of Lausanne. *Nlrc5* ^*−/−*^ and *CIIta* ^*−/−*^ were intercrossed to generate double-deficient animals. *Rfx5* ^*−/−*^ [29] and *Rfx5* ^*+/-*^ littermate controls on a mixed Sv129/C57BL/6 (H2^b^) background were bred at the animal facility of the University of Geneva Medical School. Sex and age-matched 6 to 12 week-old mice were used.

### Cells and flow cytometry

Human BLS cell lines and *in vitro* generated B cell mutants have been described and are established human cell lines [1,30]. T cells were enriched using anti-CD4 and/or anti-CD8 magnetic beads (Miltenyi Biotec). For all flow cytometric analyses, gating on living cells and exclusion of doublets was performed. Enriched TEC suspensions [2] were washed in PBS, 2% FCS, 5mM EDTA and stained for flow cytometry using death exclusion markers (either DAPI or 7AAD), UEA1 (Sigma) and the following mAb-conjugated mix: α-CD45 (30F11, BioLegend) and α-BP1 (6C3; BioLegend), α-MHCII (M5/114.15.2; eBioscience) and α-EpCAM (G8.8, Developmental Studies Hybridoma Bank, Iowa), α-H2-Db (B22/24g) and α-H2-Kb (B8.24.3). Splenocytes were preincubated with anti-CD16/32 (2.4G2) to block FcRs and stained using Abs against CD8a (Ly-2), CD3e (145-2C11), CD4 (L3T4), CD11b (M1/70), CD11c (N418), CD19 (1D3), H2-Db (28-14-8), H2-Kb (AF6–88.5.5.3), MHCII (M5/114.15.2), NK1.1 (PK136), B220 (RA3-6B2), Qa-2 (69H1-9-9) (all from eBioscience). Streptavidin conjugated to different fluorophores was from eBioscience. Stainings were performed with appropriate combinations of fluorophores. Data was acquired with a FACSCanto flow cytometer (Becton Dickinson) and analyzed using FlowJo software (Tree Star).

### ChIP-sequencing

Chromatin was purified from MACS-sorted WT (C57BL/6), *Nlrc5* ^*F/F*^, *Nlrc5* ^*−/−*^, and *Rfx5* ^*−/−*^ T cells as described [31]. Five mice were pooled for each genotype. Chromatin immunoprecipitation was performed using anti-NLRC5 antibody as described [2].

Immunoprecipitated DNA was sequenced using the Illumina HiSeq 2000 platform. >300 million reads were obtained for WT samples. >20 million reads were obtained for all other samples. ChIP samples from WT and *Nlrc5* ^*F/F*^ mice were used as biological repeats. Five pseudo-replicates of 30 million reads each were used for the WT data set, as proposed by the ENCODE consortium [32]. Reads were mapped to the mouse genome (release GRCm38.70) using Bowtie 0.12.7 [33]. Only reads mapping to unique genomic positions were considered for further analysis.

Fragment length was estimated using cross-correlation [32]. The Phantompeakqualtools R package (https://www.encodeproject.org/search/?type=software&used_by=ENCODE&software_type=quality%20metric) [32] was used to measure the quality of the ChIP-seq data, as assessed by the normalized ratio between the fragment-length cross-correlation and the background cross-correlation (normalized strand coefficient, NSC), the ratio between the fragment-length peak and the read-length peak (relative strand correlation, RSC) and the Qtag code. The low NSC scores obtained (< 1.05) (S6A Fig.) are a consequence of the low number of peaks [32]. The RSC (> 1.51–1.85) and Qtag (2, high quality) scores obtained attest to the quality of the ChIP-seq peaks (S6A Fig.) [32].

Peak calling for WT and *Nlrc5* ^*F/F*^ data sets was first done with MACS2 using the default settings (q-value threshold of 0.05 and without the “–*to-large”* parameter). This led to the identification of a surprisingly low number of reproducible peaks. The numbers of peaks were 6 and 11, respectively for the WT and *Nlrc5* ^*F/F*^ datasets. The low number of peaks called using the initial strategy prompted us to use second strategy based on using a lower peak calling stringency followed by Irreproducible Discovery Rate (IDR) analysis. This was done to ascertain that that the low number of peaks identified by our initial procedure was not in fact an artifact resulting from overly-stringent peak selection. Peaks were called using MACS2 2.0.10.20130520 [34] with no-model setting and shift-size parameter set to half of the estimated fragment length. Peak calling stringency was decreased by using p = 0.001 as threshold and applying the “-to-large” setting. Reads obtained from *Nlrc5* ^*−/−*^ samples were used as negative control for peak calling. Reproducible peaks were obtained by assessing the IDR for all pairs of pseudo-replicates using a threshold of 0.01 (S6B Fig.). Only 11 reproducible peaks were obtained, all of which were confirmed in the biological repeat (*Nlrc5* ^*F/F*^) but found to be absent in the *Rfx5* ^*−/−*^ and *Nlrc5* ^*−/−*^ samples. These 11 peaks were the same as those identified in the *Nlrc5* ^*F/F*^ dataset with the first peak identification strategy.

The Fraction of Reads in Peaks (FRiP) [32] was also calculated (S6C Fig.). The low FRiP values obtained (<1%) are consistent with the low number of peaks identified [32].

### Sequence analysis

For each gene, all annotated exons (release GRCm38.69) from all isoforms were used to create a unique gene model in which all exons were merged into a single mRNA. The TSS of this unique gene model was defined as the TSS for the corresponding gene. The promoter region was defined as the region spanning −500bp to +50bp of the TSS. Peaks overlapping with promoter regions were used for *de novo* motif discovery using the package cosmo [35] available for the R project [36]. An initial search identified a motif corresponding to the previously published X box [15]. Peaks were oriented relative to this X motif, and searches for S and Y motifs were then performed within 60 (Figs. 5, S4A, S1 Table) or 100 (S5, S4B Figs.) base-pair windows situated upstream and downstream of the center of the X box. This identified upstream and downstream motifs corresponding, respectively, to the previously described S and Y boxes [15]. Genome wide search for modules containing the 3 motifs was performed using both the Position Weight Matrix (PWM) for each motif and the minimal and maximal distances between the motifs. The consensus for each motif was represented by a PWM obtained by aligning the sequences of the corresponding motif observed in peaks. The score of each sequence versus its PWM was calculated for each peak, and 95% of this minimal score was used as threshold for the genome wide search. Authorized spacing between the motifs in the genome wide search was considered as that observed between the motifs found in peaks plus or minus 5nt. Only sequence modules containing the 3 motifs separated by the authorized distances were accepted.

### ChIP

Chromatin was purified as described [31] from *Nlrc5* ^*F/F*^, *Nlrc5* ^*−/−*^, *Rfx5* ^*−/−*^ and *Rfx5* ^*+/-*^MACS-sorted T cells (Fig. 3B, four to five mice were pooled per genotype), *Nlrc5* ^*+/−*^ *CIIta* ^*+/−*^, *Nlrc5* ^*−/−*^, *CIIta* ^*−/−*^ and *Nlrc5* ^*−/−*^ *CIIta* ^*−/−*^ B cells (Fig. 4B, two mice were pooled per genotype), or WT (C57BL/6), *Nlrc5* ^*−/−*^ and *Rfx5* ^*−/−*^ B cells (S2B Fig., four to five mice were pooled per genotype). Chromatin immunoprecipitation was performed using anti-NLRC5 and anti-CIITA antibodies as described [2,31]. Analysis of specific DNA regions was performed by qRT-PCR with the primers shown in Table 2.

**Table 2:** Primers used for qRT-PCR analysis after ChIP

Mouse Gene	Forward	Reverse
*H2-D*	CTTCTGCCGGGACACTGATGAC	GCTGATTGGCTCCTGGAATCTC
*H2-K*	TCACTTCTGCACCTAACC	GGCTGCGTGGACTTTATATC
*B2m*	TAGTAGGGCACCAAGGGTCCAG	ACTCACAGCCAATCCGGACTC
*H2-Q4*	GGGTTCAGGCAAAGTCTTAG	GTGAGAACTGCCAGTAAGTC
*H2-Q6*	AGGTGATCACCGGGAACC	GCAAGAGCAGCGTTGTTAGA
*H2-Q7*	GCAGTGAGGTCAAGGGTAG	GTGAGAACTGTTACACGTCA
*H2-T10*	CTGAGAGCCATTGCCTGGGA	CTTCTGGAGGAGTCCTGTGT
*H2-T22*	GTCCTTGCCTGCCAGGGATT	CAGACAGCCTCTGAACAGGT
*Tap1*	ATCACTCCACCCGCTGACTCTC	CGCAGCCGCTGATTGGAAAG
*H2-Eb1*	AGCAGCCCAGACTGAGTATC	GGGAGCCAATCAGCATCAAAG
*TBP*	GCTTGCTTGGGCTTGATCG	ACGGCCCTTTCTAGCTGTC
*Baseline*	TCTCTGGGCAACCTTTGTTC	CCCAGTCCTTTGGTTCTTTG

### Quantitative RT-PCR

Total RNA was extracted using the TriFast^TM^ reagent according to manufacturer's instructions (PEQLAB Biotechnologie). Retrotranscription to cDNA, quantification, and data analysis have been described [37]. Expression was determined relative the indicated housekeeping gene. Primer sequences used are listed in Table 3.

**Table 3:** Primers used for qRT-PCR analysis

**Human Gene**	**Forward**	**Reverse**
*HLA-C*	CAGAAGTACAAGCGCCAGG	TAGGCGGACTGGTCATACC
*HLA-A*	AAAAGGAGGGAGTTACACTCAGG	GCTGTGAGGGACACATCAGAG
*ACTB*	CTGGCACCCAGCACAATG	GCCGATCCACACGGAGTACT
**Mouse Gene**	**Forward**	**Reverse**
*H2-D*	ACCCAGGACATGGAGCTTGT	GCTCCAAGGACACCCAGAAC
*H2-K*	TTGAATGGGGAGGAGCTGAT	GCCATGTTGGAGACAGTGGA
*B2m*	CCTGGTCTTTCTGGTGCTTG	TTCAGTATGTTCGGCTTCCC
*Tap1*	TACCCAAACCAGCCCAAAGT	GGTGCTCTTCCCTGATCCAT
*Lmp2*	TGGTTATGTGGACGCAGCTT	ACACCCCCACTAGAGCCATC
*H2-T22*	TCGCTCTAGTTTATAAAGCTGTCCAAG	TGTAGAAATACCTAAGCGAGTGTGAAC
*H2-Q4*	AACAATGCTGCTTCTGCTGGT	GTGAGACCCCGAACTCCTTC
*H2-M3*	CTCTTCATCCTTCTGCCAGG	AGGAGATTCTTCAGCGAGCA
*Hprt*	GCAGTACAGCCCCAAAATGG	AACAAAGTCTGGCCTGTATCCAA

### Phylogenetic tree of mouse MHC molecules

The following amino-acid sequences were downloaded from MGI (http://www.informatics.jax.org/): H2-Ke2 (MGI:95908), H2-K1 (MGI:95904), H2-Ke6 (MGI:95911), H2-Oa (MGI:95924), H2-DMa (MGI:95921), H2-DMb2 (MGI:95923), H2-DMb1 (MGI:95922), H2-Ob (MGI:95925), H2-Ab1 (MGI:103070), H2-Aa (MGI:95895), H2-Eb1 (MGI:95901), H2-Eb2 (MGI:95902), H2-D1 (MGI:95896), H2-Q1 (MGI:95928), H2-Q2 (MGI:95931), H2-Q4 (MGI:95933), H2-Q6 (MGI:95935), H2-Q7 (MGI:95936), H2-Q10 (MGI:95929), H2-T24 (MGI:95958), H2-T23 (MGI:95957), H2-T22 (MGI:95956), H2-T17 (MGI:95949), H2-M10.1 (MGI:1276522), H2-T10 (MGI:95942), H2-T3 (MGI:95959), H2-M10.2 (MGI:1276525), H2-M10.4 (MGI:1276527), H2-M1 (MGI:95913), H2-M9 (MGI:1276570), H2-M10.3 (MGI:1276524), H2-M11 (MGI:2676637), H2-M10.5 (MGI:1276526), H2-M5 (MGI:95917), H2-M3 (MGI:95915), H2-M2 (MGI:95914), Mill1 (MGI:2179988), Cd1d1 (MGI:107674), B2m (MGI:88127), Mr1 (MGI:1195463), Azgp1 (MGI:103163), Mill2 (MGI:2179989), Fcgrt (MGI:103017), Cd1d2 (MGI:107675), H2-Q8 (MGI:95937), H2-Q9 (MGI:95938) and H2-T9 (MGI:95965). Alignment was performed using the Muscle tool [38], the best model to construct the phylogenetic tree was assessed using Prottest [39], and the phylogenetic tree was constructed in PhyML [40] using the JTT substitution model.

### Luciferase reporter gene assays

Luciferase reporter plasmids were created by replacing the MluI—BglII fragment spanning the HLA-DRA SXY region in the pDRAprox plasmid [15] with the corresponding *H2-K*, *H2-Eb1* and hybrid SXY regions. The pGL3-min plasmid containing only the *HLA-DRA* core promoter (from −60 to +10) in the same reporter plasmid was used as negative control. DNA fragments corresponding to the SXY regions were generated using partially complementary primers that were annealed and amplified by PCR using GoTaq polymerase (Promega). Primer sequences used are listed below. Extensions containing the MluI and BglII restriction sites (underlined) used for cloning are indicated in smaller font.

### H2-K construct:

5’ATGCACGCGTCCACAGTTTCACTTCTGCACCTAACCTGGGTCAGGTCCTTCTGTCCGGACACTGTTG 3’ (forward primer)

5’TGGTAGATCTCGCCACCCAATGGGGGTAAGAGCTGACTGCGCGTCAACAGTGTC 3’ (reverse primer)

### H2-E construct:

5’ATGCACGCGTAACTGCAAGTTTCAGAAGGGGACCTGCAAACTGAATCTCTAACTAGCAACTGATGA 3’ (forward primer)

5’TGGTAGATCTTGGGAGCCAATCAGCATCAAAGGAGTCCAGCATCATCAGTTG 3’ (reverse primer)

### Hybrid H2-K/E construct 1:

5’ATGCACGCGTAACTGCAAGTTTCAGAAGGGGACCTGGGTCAGGTCCTTCTGTCCGG ACACTGTTG 3’ (forward primer)

5’TGGTAGATCTCGCCACCCAATGGGGGTAAGAGCTGACTGCGCGTCAACAGTGTC 3’ (reverse primer)

### Hybrid H2-K/E construct 2:

5’ATGCACGCGTCCACAGTTTCACTTCTGCACCTAACCTGGGTCAGGTCCTTCTGACTAGCAACTGATGA 3’ (forward primer)

5’TGGTAGATCTCGCCACCCAATGGGGGTAAGAGCTGACTGCGCATCATCAGTTG 3’ (reverse primer)

### Hybrid H2-K/E construct 3:

5’ATGCACGCGTCCACAGTTTCACTTCTGCACCTAACCTGGGTCAGGTCCTTCTGTCCGGACACTGTTG 3’ (forward primer)

5’TGGTAGATCTTGGGAGCCAATGGGGTAAGAGCTGACTGCGCGTCAACAGTGTC3’ (reverse primer)

### Hybrid H2-K/E construct 4:

5’ATGCACGCGTCCACAGTTTCACTTCTGCACCTAACCTGCAAACTGAATCTCTAACTAGCAACTGATGA 3’ (forward primer)

5’TGGTAGATCTTGGGAGCCAATCAGCATCAAAGGAGTCCAGCATCATCAGTTG 3’ (reverse primer)

### Hybrid H2-K/E construct 5:

5’ATGCACGCGTAACTGCAAGTTTCAGAAGGGGACCTGCAAACTGAATCTCTATCCGGACACTGTTG3’ (forward primer)

5’TGGTAGATCTTGGGAGCCAATCAGCATCAAAGGAGTCCAGCGTCAACAGTGTC 3’ (reverse primer)

### Hybrid H2-K/E construct 6:

5’ATGCACGCGTAACTGCAAGTTTCAGAAGGGGACCTGCAAACTGAATCTCTAACTAGCAACTGATGA 3’ (forward primer)

5’TGGTAGATCTCGCCACCCAATGCAGCATCAAAGGAGTCCAGCATCATCAGTTG 3’ (reverse primer)

HEK293T cells were subconfluently seeded into a 96-well plate and co-transfected with Polyfect reagent (Qiagen) following the manufacturer’s instructions with 25 ng of empty, human NLRC5 or human CIITA (pIII) expression vectors and 25 ng of the indicated luciferase reporter constructs [2]. 5 ng of Renilla luciferase vector were included for normalization. Cells were harvested 22h post-transfection and cell lysates were analyzed using the Dual-Luciferase Reporter Assay System (Promega) following the manufacturer’s instructions.

### Statistical analysis

Statistical differences were calculated as described in the Figure legends. Differences were considered significant when p≤0.05 (*), very significant when p≤0.01 (**) and extremely significant when p≤0.001 (***).

### Ethics statement

Mice were treated in accordance with the Swiss Federal Veterinary Office guidelines. Human cell lines are established cell lines.

## Supporting Information

S1 Fig T cell homeostasis and further analysis of the MHC phenotype in *Nlrc5^−/−^*, *CIIta^−/−^* and *Nlrc5^−/−^*CIIta*^−/−^* mice. (A) Graphs depict the MFIs of H2-D for CD8^+^ T cells (gated as CD3^+^CD8^+^), CD4^+^ T cells (gated as CD3^+^CD4^+^), NK cells (gated as NK1.1^+^CD3^-^), NKT cells (gated as NK1.1^+^CD3^+^), B cells (gated as CD19^+^), and DCs (gated as CD11c^hi^CD11b^int-hi^) from control (n = 6), *Nlrc5* ^−/−^ (n = 6), *CIIta* ^−/−^ (n = 6) and double-deficient (n = 5) mice. Results depict the mean ± SEM from two pooled experiments, and are representative of three independent experiments. Differences among multiple groups were analyzed by 2-way ANOVA adjusted by Bonferroni correction over 6 samples and are shown only for the effect double as compared to single-deficiency. (B) MFIs for H2-K and H2-D expression by *Nlrc5* ^*−/−*^ (n = 4 mice), *CIIta* ^*−/−*^ (n = 4 mice), and *Nlrc5* ^*−/−*^ *CIIta* ^*−/−*^ (n = 4 mice) mTECs (gated as CD45^*−*^ UEA1^+^) are expressed as percentage of *Nlrc5* ^*+/−*^ *CIIta* ^*+/−*^ (n = 4 mice) control mice mTECs. Results represent the mean ± SEM derived from two pooled experiments, and are representative of three independent experiments. The histogram overlay for MHCII expression shows a representative mouse for each group. Differences among multiple groups were analyzed by 2-way ANOVA and are shown only for the effect double as compared to single-deficiency. (C) Percentages and numbers of splenic CD8^+^ (gated as CD3^+^CD8^+^) and CD4^+^ T cells (gated as CD3^+^CD4^+^) from control (n = 6), *Nlrc5* ^−/−^ (n = 6), *CIIta* ^−/−^ (n = 6) and *Nlrc5* ^*−/−*^ *Ciita* ^*−/−*^ (n = 5) mice are shown. Results depict the mean ± SEM from two pooled experiments, and are representative of three independent experiments.
(TIF)

S2 Fig Characterization of NLRC5 targets. (A) NLRC5-ChIPseq tracks are shown for the NLRC5 targets *B2m* and *Psmb9/Tap1*. The tracks depict reads mapping to regions spanning between 5kb upstream and 5kb downstream of the TSS. After normalization as rpm (reads per million), read coverage was expressed relative to the maximal value observed in the region. TSSs are positioned as annotated in Refseq (*Psmb9*) or ENSEMBL (others). (B) Antibodies specific for NLRC5 were used to immunoprecipitate cross-linked chromatin fragments derived from WT, *Nlrc5* ^*−/−*^ and *Rfx5* ^*−/−*^ B cells. Immunoprecipitates were analyzed by quantitative PCR for the abundance of promoter sequences from the indicated genes. Relative promoter binding is shown. Results represent the average ± SD of technical triplicates.
(TIF)

S3 Fig Schematic alignment of the promoter regions of mouse NLRC5 target genes. Positions of the SXY modules and distal regulatory elements (interferon stimulation response element (ISRE) and enhancer A) defined in classical MHCI promoters are indicted by colored boxes. Predicted motifs were identified by homology searches on the basis the sequence of the upper (boxes above line) and/or or lower (boxes below line) strands.
(TIF)

S4 Fig Spacing requirements for the recruitment of NLRC5 to SXY sequence modules. S-X and X-Y spacing distributions are shown for SXY modules occupied by NLRC5 (top graphs) and all genomic matches to the SXY consensus module (bottom graphs) defined by two different motif discovery approaches giving more (A, maximum 60 base pairs) or less (B, maximum 100 bp) weight to S-X and X-Y spacing. The percent of modules (Y axis) are plotted as a function of distance in base pairs (X axis) between the S and X motifs (red bars) or the X and Y motifs (blue bars). The S, X and Y sequence logos are shown below. The most prominent spacing characteristic of NLRC5-occupied modules are indicated at the top and highlighted by red and blue underlays.
(TIF)

S5 Fig Identification of a consensus motif for NLRC5 recruitment using a less stringent spacing constraint. Alignment of sequence motifs situated within NLRC5-occupied peaks found in promoter regions of the indicated genes. S-X and X-Y distance constraints used for identifying the S and Y motifs were set at a maximum of 100 base pairs for each. Distances (bp) between motifs are indicated. The sequence logo for the consensus NLRC5-module is shown below the alignment and is compared with that previously defined for human CIITA.
(TIF)

S6 Fig ChIP-seq metrics. (A) Strand cross-correlation analysis was performed for 5 WT pseudo-replicates (A_WT, B_WT, C_WT, D_WT, E_WT), the complete WT dataset (All_WT), and the *Nlrc5* ^*F/F*^ dataset. Peak heights corresponding to the average fragment size (dashed blue lines) are markedly higher than peak heights corresponding to read length (first dashed red lines to the right of the dashed blue lines). NSC, RSC, and Qtag values are indicated below each graph. Low NSC values are expected due to the low number of peaks. RSC and Qtag values attest to the quality of the ChIP-seq peaks. (B) Irreproducible Discovery Rate (IDR) analysis on pseudo-replicates. Coloured lines represent all pairwise comparisons between the 5 WT pseudo-repeats. Peaks were called with the MACS2 peak caller using p<0.001 and the “–to-large” setting. Numbers of called peaks before IDR analyses were 459, 529, 1436, 1043 and 1207 respectively. The IDR threshold was set at 0.01. (C) Fraction of Reads in Peaks (FRiP) values. Left panel: FRiP values are represented for each WT pseudo-replicate, before and after IDR analysis. Numbers of peaks called in each pseudo-replicate before IDR analyses were as in B. The number of final peaks (after IDR analysis) was 11. Right panel: FRiP values are shown for each pseudo-replicate and the original WT data set when the peak calling procedure was performed using the default parameters of MACS2. Numbers of called peaks were 11, 11, 11, 11, 10 and 6 for the pseudo-replicates and the complete WT dataset, respectively. Low FRiP values are expected due to the low number of peaks.
(TIF)

S1 Table Genes having a predicted SXY module in their promoter region. (DOCX)

S2 Table Gene location in ENSEMBL and UCSC databases.
