# Supplementary figures and images for: NLRC5 Exclusively Transactivates MHC Class I and Related Genes through a Distinctive SXY Module
Source: PLoS Genet. 2015 Mar 26;11(3):e1005088. doi: 10.1371/journal.pgen.1005088 (PMC4374748; doi:10.1371/journal.pgen.1005088)

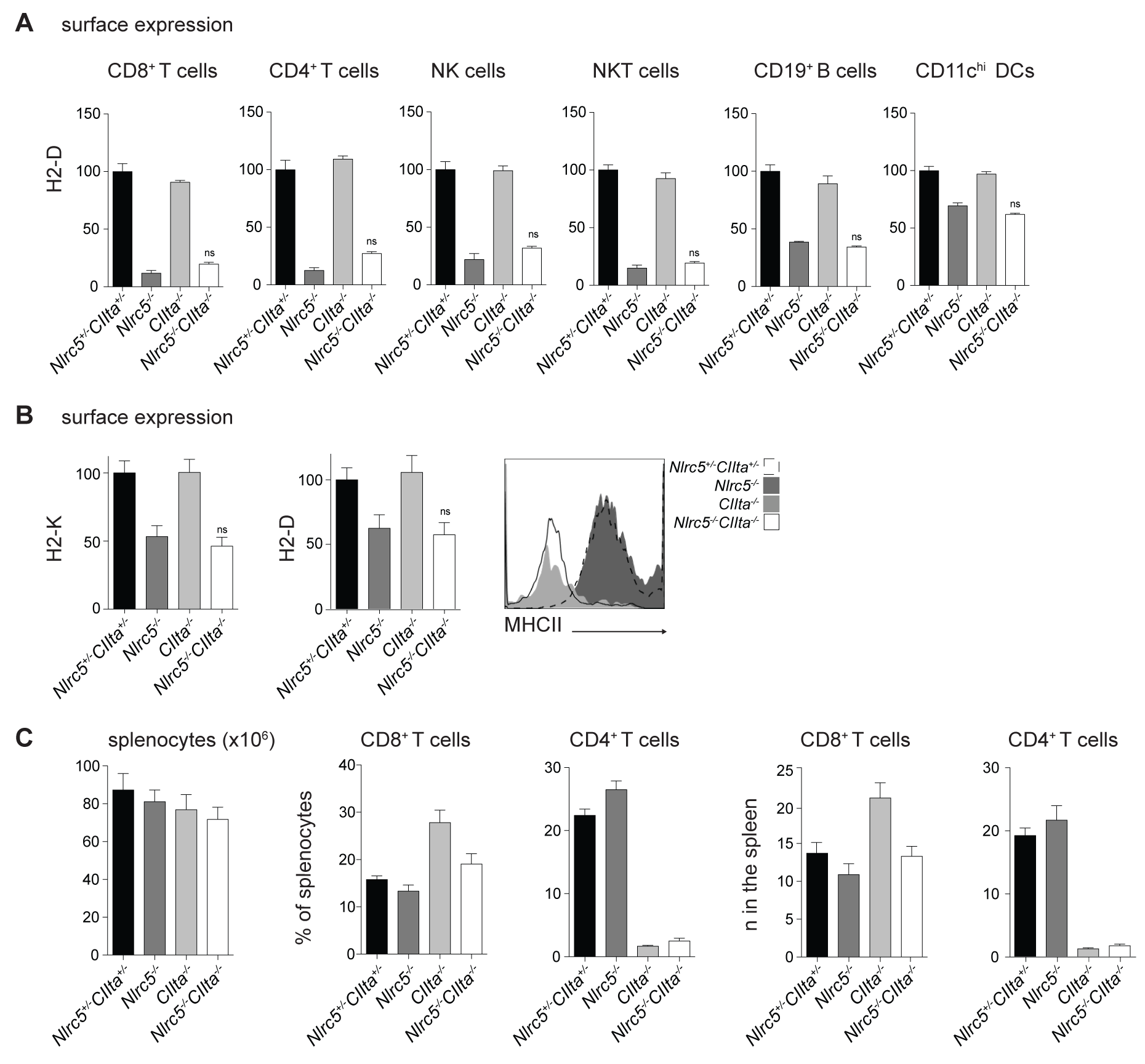

Supplement: S1 Fig — (A) Graphs depict the MFIs of H2-D for CD8+ T cells (gated as CD3+CD8+), CD4+ T cells (gated as CD3+CD4+), NK cells (gated as NK1.1+CD3-), NKT cells (gated as NK1.1+CD3+), B cells (gated as CD19+), and DCs (gated as CD11chiCD11bint-hi) from control (n = 6), Nlrc5 −/− (n = 6), CIIta −/− (n = 6) and double-deficient (n = 5) mice. Results depict the mean ± SEM from two pooled experiments, and are representative of three independent experiments. Differences among multiple groups were analyzed by 2-way ANOVA adjusted by Bonferroni correction over 6 samples and are shown only for the effect double as compared to single-deficiency. (B) MFIs for H2-K and H2-D expression by Nlrc5 −/− (n = 4 mice), CIIta −/− (n = 4 mice), and Nlrc5 −/− CIIta −/− (n = 4 mice) mTECs (gated as CD45− UEA1+) are expressed as percentage of Nlrc5 +/− CIIta +/− (n = 4 mice) control mice mTECs. Results represent the mean ± SEM derived from two pooled experiments, and are representative of three independent experiments. The histogram overlay for MHCII expression shows a representative mouse for each group. Differences among multiple groups were analyzed by 2-way ANOVA and are shown only for the effect double as compared to single-deficiency. (C) Percentages and numbers of splenic CD8+ (gated as CD3+CD8+) and CD4+ T cells (gated as CD3+CD4+) from control (n = 6), Nlrc5 −/− (n = 6), CIIta −/− (n = 6) and Nlrc5 −/− Ciita −/− (n = 5) mice are shown. Results depict the mean ± SEM from two pooled experiments, and are representative of three independent experiments. (TIF) [file pgen.1005088.s001.tif]

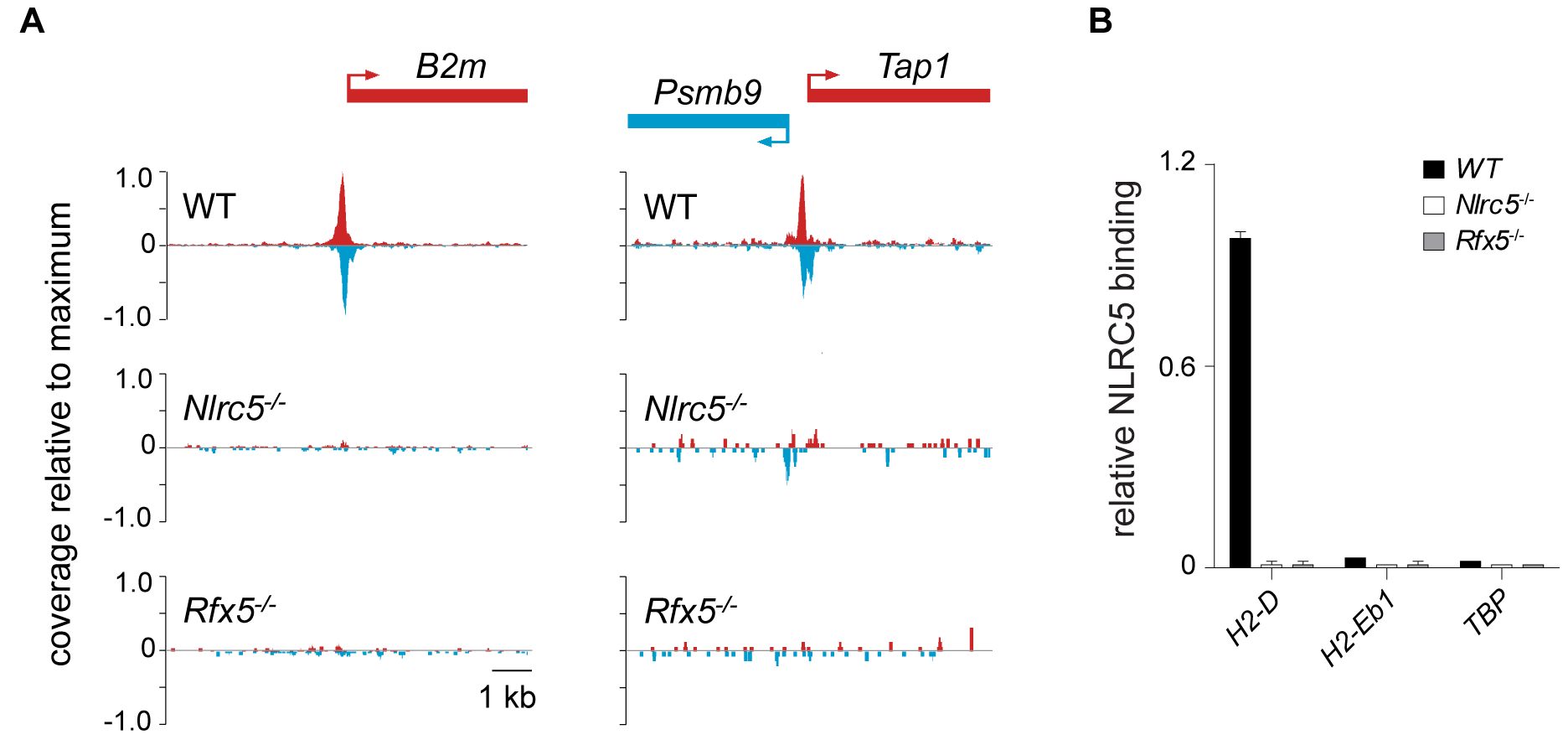

Supplement: S2 Fig — (A) NLRC5-ChIPseq tracks are shown for the NLRC5 targets B2m and Psmb9/Tap1. The tracks depict reads mapping to regions spanning between 5kb upstream and 5kb downstream of the TSS. After normalization as rpm (reads per million), read coverage was expressed relative to the maximal value observed in the region. TSSs are positioned as annotated in Refseq (Psmb9) or ENSEMBL (others). (B) Antibodies specific for NLRC5 were used to immunoprecipitate cross-linked chromatin fragments derived from WT, Nlrc5 −/− and Rfx5 −/− B cells. Immunoprecipitates were analyzed by quantitative PCR for the abundance of promoter sequences from the indicated genes. Relative promoter binding is shown. Results represent the average ± SD of technical triplicates. (TIF) [file pgen.1005088.s002.tif]

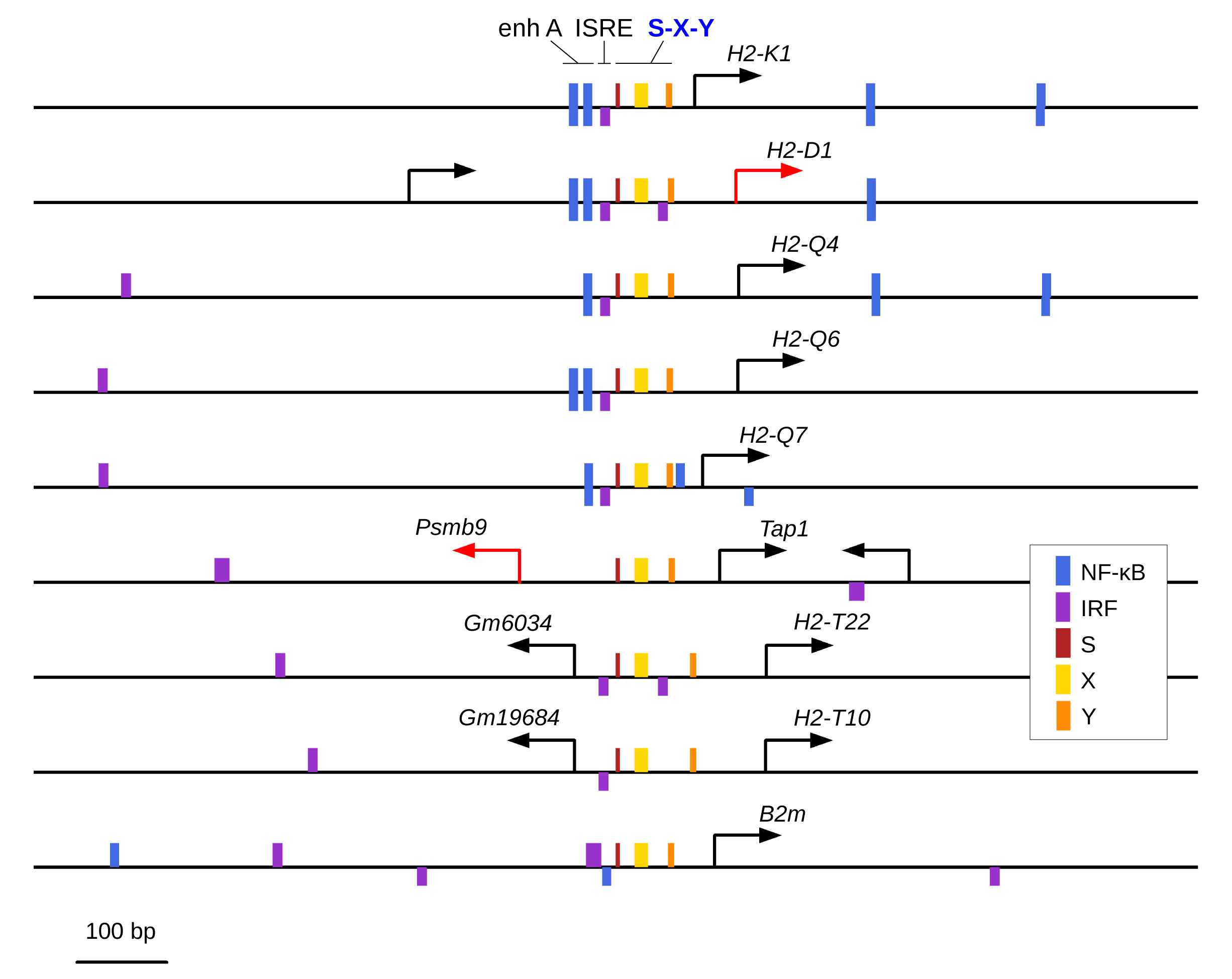

Supplement: S3 Fig — Positions of the SXY modules and distal regulatory elements (interferon stimulation response element (ISRE) and enhancer A) defined in classical MHCI promoters are indicted by colored boxes. Predicted motifs were identified by homology searches on the basis the sequence of the upper (boxes above line) and/or or lower (boxes below line) strands. (TIF) [file pgen.1005088.s003.tif]

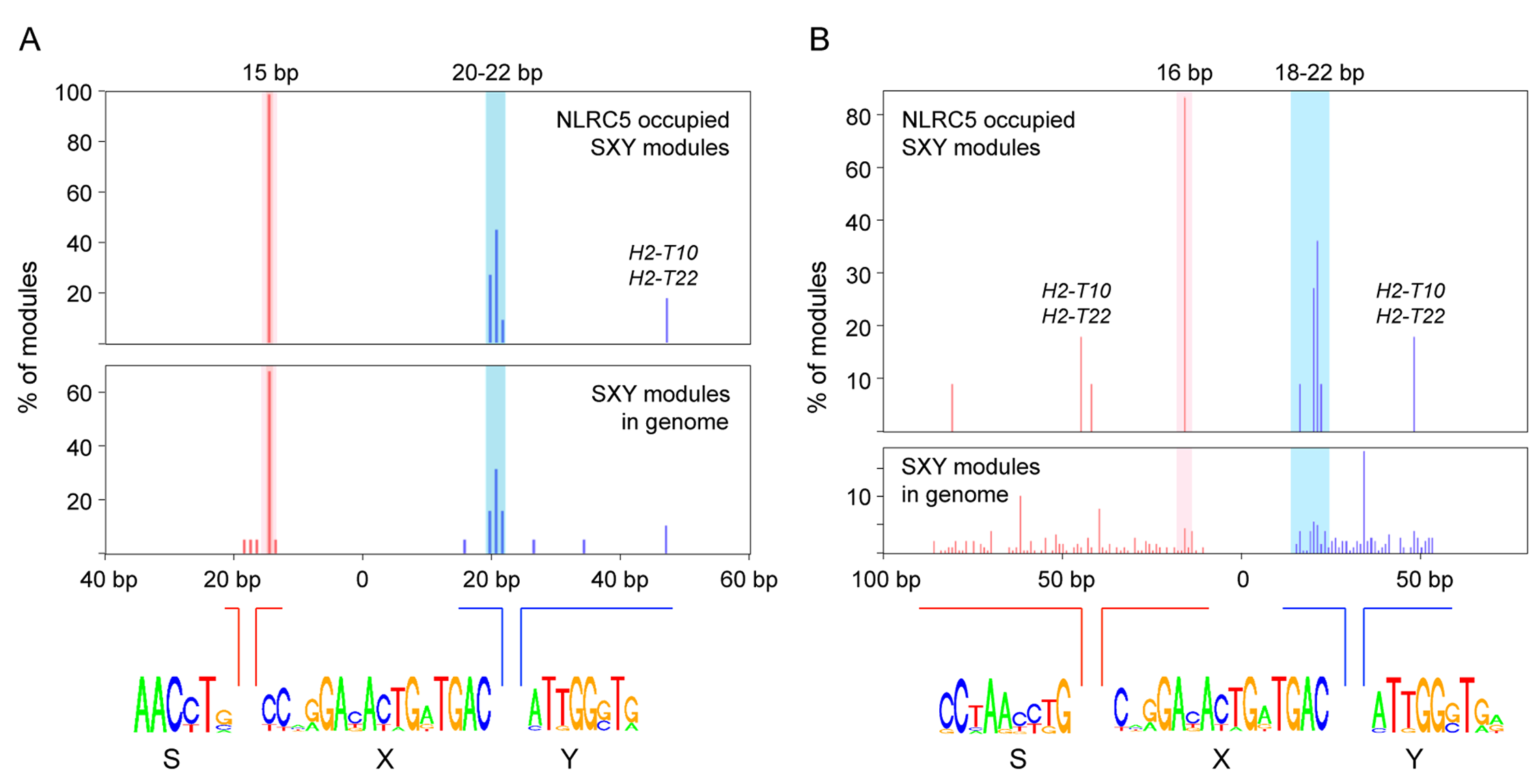

Supplement: S4 Fig — S-X and X-Y spacing distributions are shown for SXY modules occupied by NLRC5 (top graphs) and all genomic matches to the SXY consensus module (bottom graphs) defined by two different motif discovery approaches giving more (A, maximum 60 base pairs) or less (B, maximum 100 bp) weight to S-X and X-Y spacing. The percent of modules (Y axis) are plotted as a function of distance in base pairs (X axis) between the S and X motifs (red bars) or the X and Y motifs (blue bars). The S, X and Y sequence logos are shown below. The most prominent spacing characteristic of NLRC5-occupied modules are indicated at the top and highlighted by red and blue underlays. (TIF) [file pgen.1005088.s004.tif]

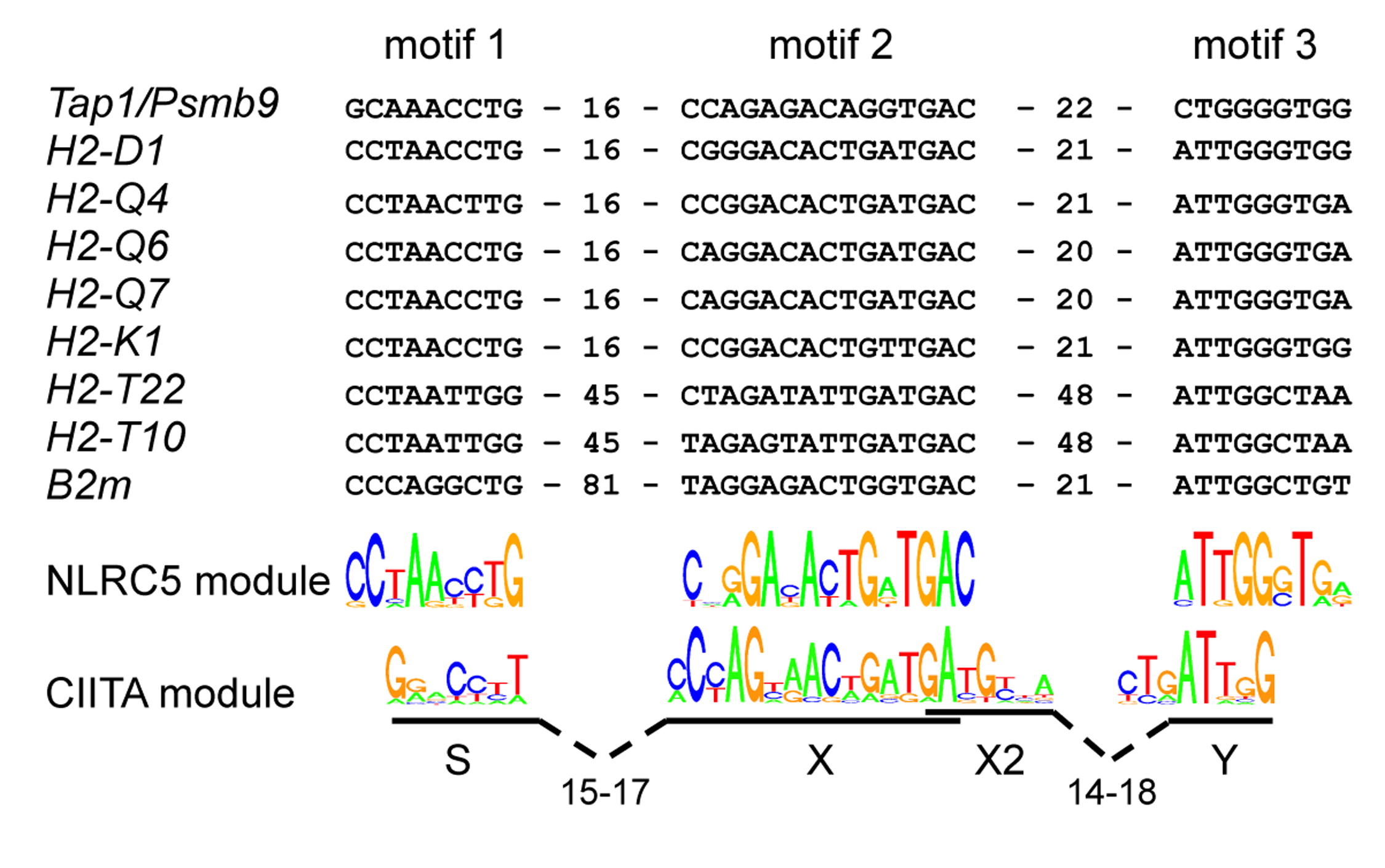

Supplement: S5 Fig — Alignment of sequence motifs situated within NLRC5-occupied peaks found in promoter regions of the indicated genes. S-X and X-Y distance constraints used for identifying the S and Y motifs were set at a maximum of 100 base pairs for each. Distances (bp) between motifs are indicated. The sequence logo for the consensus NLRC5-module is shown below the alignment and is compared with that previously defined for human CIITA. (TIF) [file pgen.1005088.s005.tif]

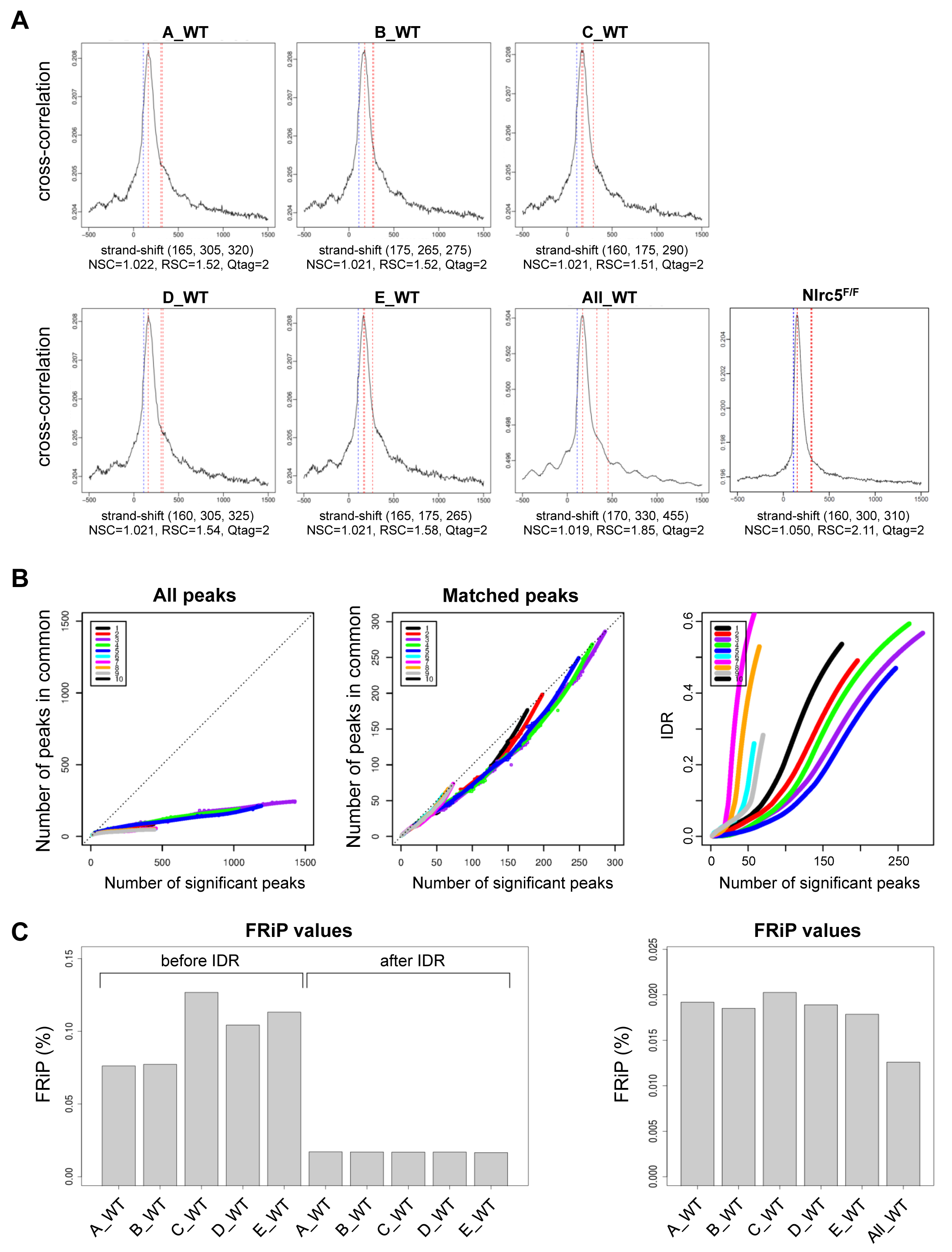

Supplement: S6 Fig — (A) Strand cross-correlation analysis was performed for 5 WT pseudo-replicates (A_WT, B_WT, C_WT, D_WT, E_WT), the complete WT dataset (All_WT), and the Nlrc5 F/F dataset. Peak heights corresponding to the average fragment size (dashed blue lines) are markedly higher than peak heights corresponding to read length (first dashed red lines to the right of the dashed blue lines). NSC, RSC, and Qtag values are indicated below each graph. Low NSC values are expected due to the low number of peaks. RSC and Qtag values attest to the quality of the ChIP-seq peaks. (B) Irreproducible Discovery Rate (IDR) analysis on pseudo-replicates. Coloured lines represent all pairwise comparisons between the 5 WT pseudo-repeats. Peaks were called with the MACS2 peak caller using p<0.001 and the “–to-large” setting. Numbers of called peaks before IDR analyses were 459, 529, 1436, 1043 and 1207 respectively. The IDR threshold was set at 0.01. (C) Fraction of Reads in Peaks (FRiP) values. Left panel: FRiP values are represented for each WT pseudo-replicate, before and after IDR analysis. Numbers of peaks called in each pseudo-replicate before IDR analyses were as in B. The number of final peaks (after IDR analysis) was 11. Right panel: FRiP values are shown for each pseudo-replicate and the original WT data set when the peak calling procedure was performed using the default parameters of MACS2. Numbers of called peaks were 11, 11, 11, 11, 10 and 6 for the pseudo-replicates and the complete WT dataset, respectively. Low FRiP values are expected due to the low number of peaks. (TIF) [file pgen.1005088.s006.tif]
