# Supplementary material for: NLRC5 Exclusively Transactivates MHC Class I and Related Genes through a Distinctive SXY Module
Source: PLoS Genet. 2015 Mar 26;11(3):e1005088. doi: 10.1371/journal.pgen.1005088 (PMC4374748; doi:10.1371/journal.pgen.1005088)
Supplement: S1 Table — (DOCX) [file pgen.1005088.s007.docx]

**Supplementary Table 1:** Genes having a predicted SXY module in their promoter region.

| **ENSEMBL Gene ID** | ***Gene*** | **Description** | **NLRC5 binding^1^** |
| --- | --- | --- | --- |
| ENSMUSG00000035929 | *H2-Q4* | histocompatibility 2, Q region locus 4 [Source:MGI Symbol;Acc:MGI:95933] | + |
| ENSMUSG00000037321 | *Tap1* | transporter 1, ATP-binding cassette, sub-family B (MDR/TAP) [Source:MGI Symbol;Acc:MGI:98483] | + |
| ENSMUSG00000055413 | *H2-Q5* | histocompatibility 2, Q region locus 5 [Source:MGI Symbol;Acc:MGI:95934] | + |
| ENSMUSG00000056116 | *H2-T22* | histocompatibility 2, T region locus 22 [Source:MGI Symbol;Acc:MGI:95956] | + |
| ENSMUSG00000060550 | *H2-Q7* | histocompatibility 2, Q region locus 7 [Source:MGI Symbol;Acc:MGI:95936] | + |
| ENSMUSG00000060802 | *B2m* | beta-2 microglobulin [Source:MGI Symbol;Acc:MGI:88127] | + |
| ENSMUSG00000061232 | *H2-K1* | histocompatibility 2, K1, K region [Source:MGI Symbol;Acc:MGI:95904] | + |
| ENSMUSG00000067203 | *H2-K2* | histocompatibility 2, K region locus 2 [Source:MGI Symbol;Acc:MGI:95906] | + |
| ENSMUSG00000073409 | *H2-Q6* | histocompatibility 2, Q region locus 6 [Source:MGI Symbol;Acc:MGI:95935] | + |
| ENSMUSG00000073411 | *H2-D1* | histocompatibility 2, D region locus 1 [Source:MGI Symbol;Acc:MGI:95896] | + |
| ENSMUSG00000079491 | *H2-T10* | histocompatibility 2, T region locus 10 [Source:MGI Symbol;Acc:MGI:95942] | + |

^1^ Determined by peak calling and verified by visual inspection of ChIP-seq data.
