# Supplementary material for: NLRC5 Exclusively Transactivates MHC Class I and Related Genes through a Distinctive SXY Module
Source: PLoS Genet. 2015 Mar 26;11(3):e1005088. doi: 10.1371/journal.pgen.1005088 (PMC4374748; doi:10.1371/journal.pgen.1005088)
Supplement: S2 Table [file pgen.1005088.s008.docx]

**Supplementary Table 2:** Gene location in ENSEMBL and UCSC databases

| ***Gene*** | **ENSEMBL (GRcm38.70)** | **Refseq** |
| --- | --- | --- |
| *B2m* | chr2:122147686-122153083 (+) | chr2:122147687-122153082 (+) |
| *H2-D1** | chr17:35262730-35267499 (+) | chr17:35263094-35267497 (+) |
| *H2-K1* | chr17:33996017-34000333 (-) | chr17:33996012-34000333 (-) |
| *H2-Q4* | chr17:35379617-35385290 (+) | chr17:35379617-35384674 (+) |
| *H2-Q6* | chr17:35424850-35430055 (+) | chr17:35424877-35428361 (+) |
| *H2-Q7* | chr17:35439155-35443773 (+) | chr17:35439155-35443773 (+) |
| *H2-T10* | chr17:36115876-36121465 (-) | chr17:36115876-36121435 (-) |
| *H2-T22* | chr17:36037128-36042747 (-) | chr17:36038409-36042702 (-) |
| *Psmb9** | chr17:34181988-34187764 (-) | chr17:34182099-34187330 (-) |
| *Tap1* | Chr17:34187553-34197225 (+) | chr17:34187556-34197225 (+) |

*Genes for which TSS positions (underlined) annotated in ENSEMBL and Refseq differ by >50 nucleotides; the SXY module in these genes would be situated upstream of the Refseq TSS but downstream of the ENSEMBL TSS.
